# Intra- and intermolecular interaction of anthracene moieties in 7,8-disilabicyclo[3.3.0]octadienyl-bridged bisanthracenes†

**DOI:** 10.1039/c8ra05293j

**Published:** 2018-07-13

**Authors:** Yuichiro Tokoro, Nobuhiko Ohtsuka, Shin-ichi Fukuzawa, Toshiyuki Oyama

**Affiliations:** Department of Advanced Materials Chemistry, Faculty of Engineering, Yokohama National University 79-5 Tokiwadai Hodogaya-ku, Yokohama 240-8501 Japan tokoro-yuichirou-zv@ynu.ac.jp oyama-toshiyuki-wz@ynu.ac.jp; Department of Applied Chemistry, Institute of Science and Engineering, Chuo University 1-13-27 Kasuga, Bunkyo-ku Tokyo 112-8551 Japan

## Abstract

Ruthenium-catalyzed dimerization of 9-anthrylarylsilanes afforded air-stable V-shaped bisanthracenes bridged by a 7,8-disilabicyclo[3.3.0]octadiene moiety. The intra- and intermolecular proximity of the anthracene moieties were determined by single-crystal X-ray analysis. Absorption and emission maxima of the disilabicyclo[3.3.0]octadienyl-bridged bisanthracenes in the solution state were observed at longer wavelengths than those of 9-anthryldimethylsilane and bis(9-anthryl)dimethylsilane. The V-shaped bisacenes in the solid state showed excimer emissions with moderate quantum yields.

## Abstract 2

V-shaped 9-anthrylarylsilane dimers prepared through ruthenium-catalyzed C–H cleavage showed excimer emission and bathochromic shifts by promoted contact of π-orbitals.

## Graphical abstract

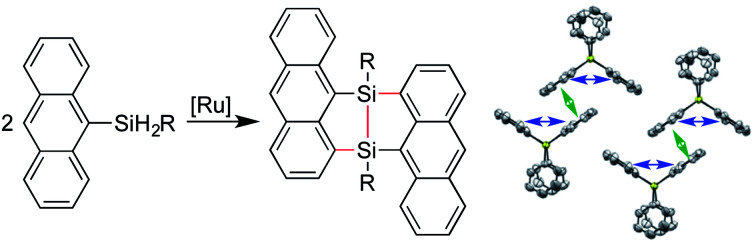

Interactions between acenes in organic materials are important for designing functions related to charge transport and photo-excitation.^1^ Anthracenes usually show strong luminescence in the dilute solution state while non-radiative deactivation of the excited state is often promoted in the solid state mainly due to polymeric π–π stacking. Crystals containing spatially isolated dimers, however, have been known to exhibit strong excimer fluorescence.^2^ In particular, a V-shape arrangement of two anthryl groups separated by *m*-phenylene has been reported to be effective for isolating dimers in luminescent crystals.^3^ Moreover, V-shaped bisacenes tightly fixed by a bicyclo[2.2.1]heptane moiety are useful for singlet fission which can improve the efficiency of solar cells.^4^ Accordingly, it is desirable to develop easily accessible bridged structures for tightly fixed bisacenes. Chernyshev *et al.* reported a V-shaped bisnaphthalene bridged by a 7,8-disilabicyclo[3.3.0]octadienyl moiety.^5^ The disilane was generated by pyrolysis of the dichloro-1-naphthylsilane at 670–690 °C and easily converted into the siloxane form (eqn 1). Bickelhaupt *et al.* described a reaction between 1,8-dilithionaphthalene and 1,1,2,2-tetrachloro-1,2-dimethylsilane to afford a similar air-sensitive V-shaped bisnaphthalene (eqn 2).^6^ Improvements to the harsh preparation conditions and air-sensitivity are required in order to investigate the utility of the 7,8-disilabicyclo[3.3.0]octadienyl-bridged bisacenes. Recently, we have developed ruthenium-catalyzed annulation of hydrosilylanthracenes with internal alkynes through Si–H and C–H bonds cleavage.^7^ The catalytic cycle of the annulation probably involves a five-membered ruthenacycle composed of ruthenium, silicon and three carbons from the anthracene moiety. Insertion of alkynes into the ruthenacycles followed by reductive elimination affords the annulation products. Herein, we modified the ruthenium catalyzed reaction for synthesis of the 7,8-disilabicyclo[3.3.0]octadienyl-bridged bisanthracenes by using dihydrosilyl anthracenes as reactants. Instead of internal alkynes, using cyclooctene as a hydrogen acceptor may moderately inhibit coordination-insertion and allow for reaction between the ruthenacycle and 9-anthrylarylsilanes to afford the V-shaped bisacenes.1
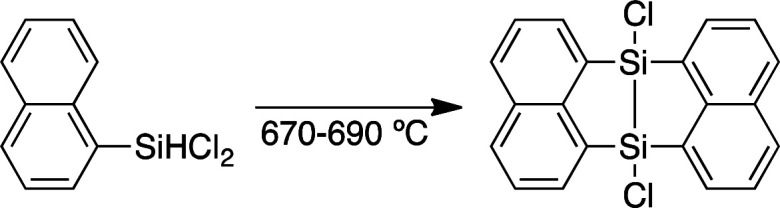
2
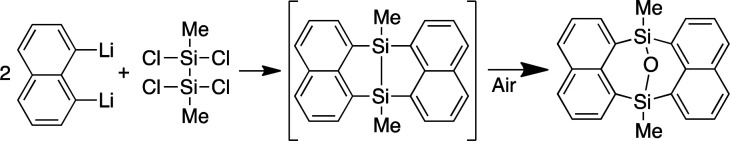
3
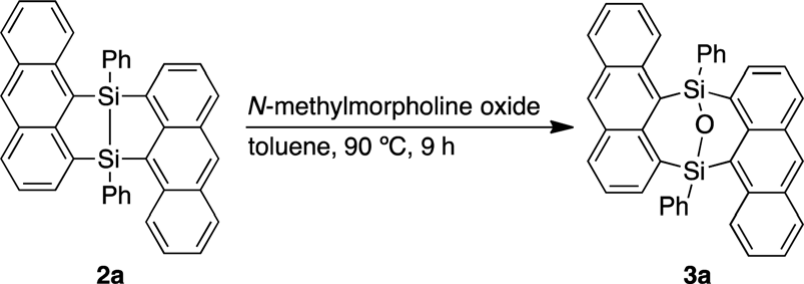


Dimerization of 9-anthrylphenylsilane proceeded in cyclopentyl methyl ether (CPME) at 115 °C (Scheme 1), under the presence of catalytic amounts of [RuH_2_(CO) (PPh_3_)_3_] and two equivalents of cyclooctene (COE). Dimer 2a was obtained as a precipitate and isolated in 39% yield by filtration of the reaction mixture. Substrates with 4-*tert*-butylphenyl (2b), 4-methoxyphenyl (2c), and 3-methoxyphenyl (2d) groups at the silicon centers were tolerated under the reaction conditions. Dimer 2a could be handled in air and no significant change in the ^1^H NMR spectrum was observed under air at 80 °C over 16 h. Oxidation of 2a, however, proceeded smoothly with *N*-methylmorpholine oxide (NMO), affording siloxane 3a through Si–Si cleavage (eqn 3). Dimer 2a also showed high thermal stability under N_2_. The degradation temperature at 5%weight loss was 401 °C (Fig. S1†).

**Scheme 1:**
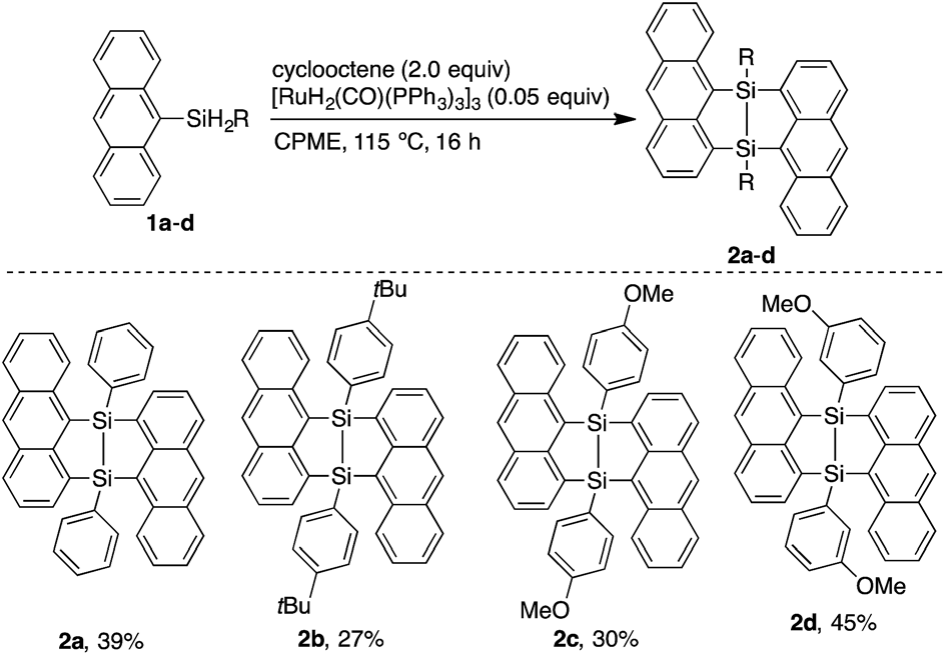
Ruthenium-catalyzed dimerization of 9-anthrylarylsilanes.

The V-shaped structure of 2a was determined by single crystal structure analysis (Fig. 1). The structure of the 7,8-disilabicyclo[3.3.0]octadienyl moiety in 2a was similar to that in bisnaphthalenes. The Si–Si and Si–C bond lengths of 2a were typical values for their single bonds. Steric restraint from the two anthracene moieties could be observed in the bond angles. The angles around the silicon center were not near the 109° of ideal sp^3^-hybridization but close to 120° and 90° corresponding to ideal sp^2^-hybridization. Distances between the 1- and 9′-position carbons were 3.125 and 3.129 Å, indicating that the π-orbitals of the anthracene moieties interacted intramolecularly through space. In the packed structure, two molecules of 2a were stacked by π–π and CH/π interactions. The interplanar distance between the π–π stacked anthracenes was 3.689 Å and the stacked anthracene pair was spatially isolated from the other pairs. The V-shaped conformation was preserved after oxidation by NMO, but the relative positions of the anthracene moieties were influenced by the insertion of oxygen between the silicon centers. The bond angles around the silicon centers came close to 109°. In particular, the C(3)–Si(1)–C(29) and C(15)–Si(2)–C(17) angles of 3a (108.77° and 108.99°, respectively) were narrower than those of 2a (112.07°), which induced closer contact of the anthracene moieties with 3.055 and 3.063 Å as the distances between carbons of 1- and 9′-positions. The average and maximum dihedral angles of 3a between the anthracene rings and planes containing C(3)–Si(1)–C(29) or C(15)–Si(2)–C(17) in 3a were 76.3° and 81.6°, respectively, while those angles in 2a were 84.8° and 88.3°. The smaller dihedral angles of 3a means it has a more twisted conformation of 3a. Although insertion of oxygen made the distance between silicon centers (2.741 Å) longer as compared with the typical Si–Si single bond length, some interaction between them probably remains. As observed in 2a, π–π stacked anthracene dimer units were observed and spatially isolated from each other.

**Fig. 1:**
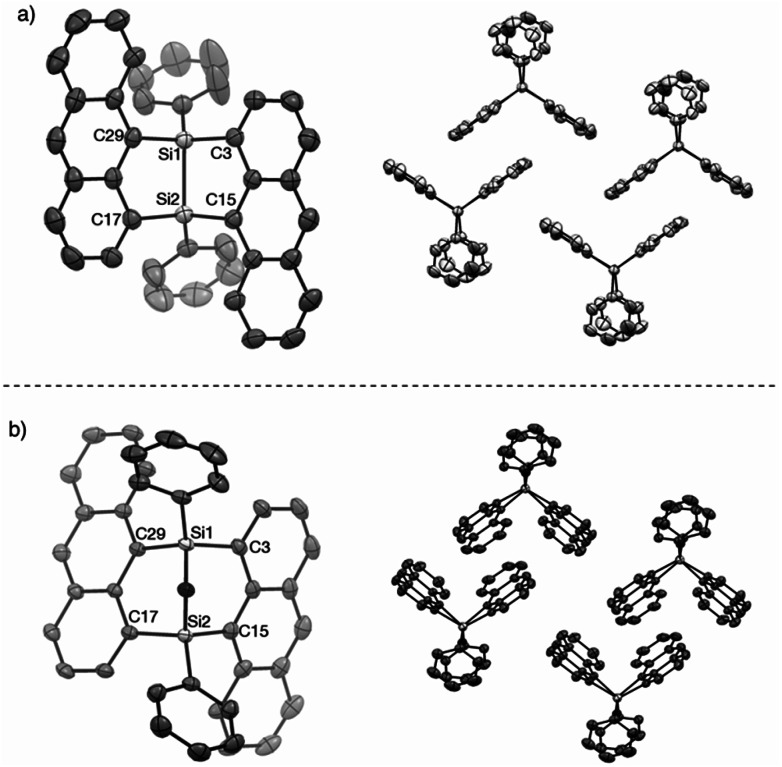
X-ray crystal structures of (a) 2a (*P*1̄) and (b) 3a (*P*1̄). Thermal ellipsoids are set to 50% probability level. Hydrogen atoms are omitted for clarity.

To elucidate the intramolecular interaction between anthracene moieties, the UV-vis spectrum of 2a in chloroform was compared with those of 9-anthryldimethylsilane (ADMS) and bis(9-anthryl)dimethylsilane (BADMS) (Fig. 2). The absorption maxima of BADMS (395, 374 and 355 nm) were observed at slightly longer wavelengths than those of ADMS (390, 369 and 351 nm), indicating that the intramolecular interaction between anthracene moieties in BADMS was weak due to easy rotation about the Si–C single bonds. In contrast, 2a showed absorption maxima at further longer wavelength region (411, 389 and 368 nm). The rigid 7,8-disilabicyclo[3.3.0]octadienyl-bridge tightly fixed the relative position of the anthracene moieties to enhance the intramolecular interaction between them. Almost no absorption peaks were shifted by substituents on the silicon centers suggested that absorption about 400 nm of the bisanthracene derived only from the anthracene moieties. Although a bathochromic shift was also observed in 3a, the shift was smaller than that of 2a. The twisted conformation of 3a may weaken the interaction between the anthracene moieties.

**Fig. 2:**
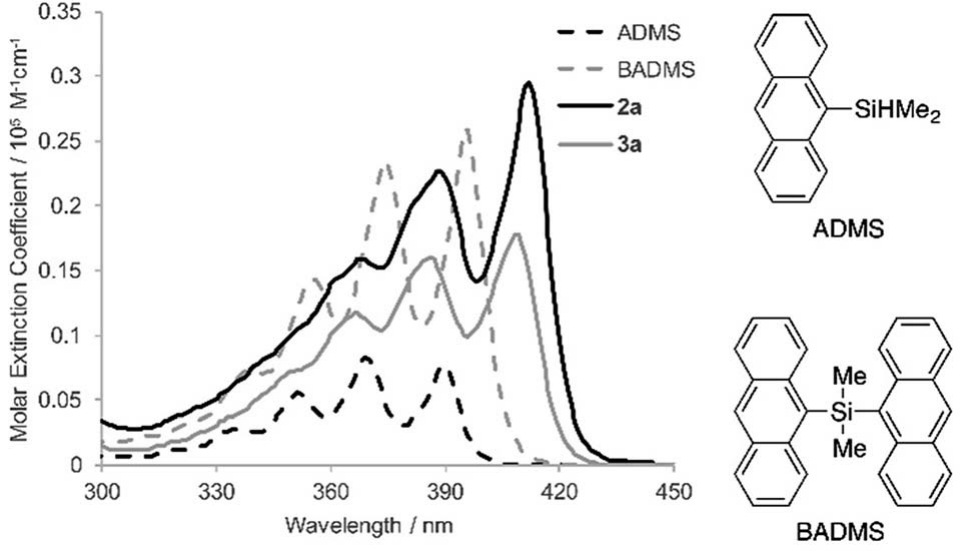
UV-vis spectra of ADMS, BADMS, 2a and 3a in chloroform (10 μM).

In the photoluminescence spectra of the chloroform solution (Fig. 3a), a similar tendency of bathochromic shifts can be seen. The V-shaped molecules 2a and 3a showed emission maxima at longer wavelengths than ADMS and BADMS. While the absolute photoluminescence quantum yields of ADMS and BADMS were 0.50 and 0.40, respectively, that of 2a decreased to 0.10. Oxidation of 2a by NMO, however, recovered the high quantum yield. The results implied that the low quantum yield of 2a is not due to the V-shape but due to the Si–Si single bond. Moreover, substituents on the silicon centers influenced the quantum yields of the disilabicyclo[3.3.0]octadienyl-bridged bisacenes. While 4-*tert*-butylphenyl and 4-methoxyphenyl groups decreased the quantum yields, 3-methoxyphenyl group increased it. The substituent effects suggested π-donating substituents on the silicon centers promoted non-radiative relaxation.

**Fig. 3:**
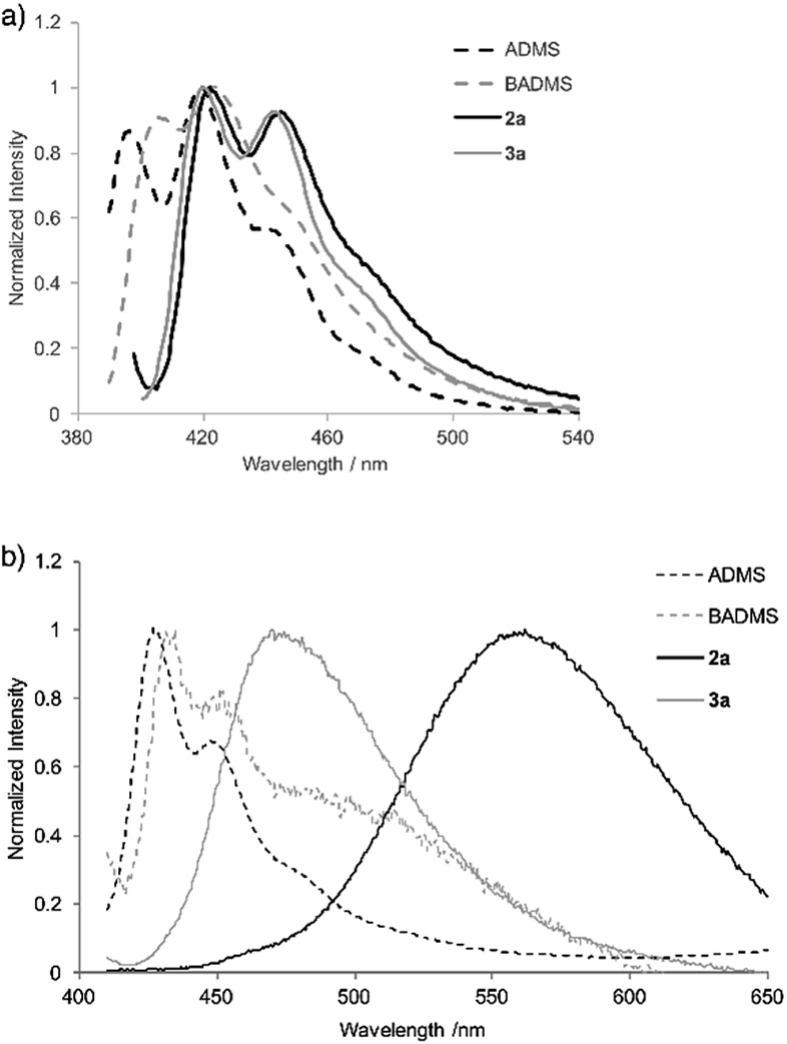
Photoluminescence spectra of ADMS, BADMS, 2a and 3a (a) in chloroform (10 μM) and (b) in powder state.

Photoluminescence of 2a in the solid state was drastically changed as compared with that in the solution state (Fig. 3b). The emission peak was observed at 562 nm with moderate quantum yield (*Φ*_F_ = 0.10) while the emission maxima of ADMS and BADMS in solid state were observed at 427 and 435 nm, respectively. The large bathochromic shift of luminescence from 2a also appeared in suspension state by THF/water (1/99; v/v, Fig. S4†). An excitation spectrum (Em = 490 nm) of 2a in the suspension state was almost coincided with the UV-vis spectrum of 2a in the chloroform solution state. The similarity of the excitation and UV-vis spectra indicated that excitation of electronically monomeric anthracene moieties in the solid and suspension states immediately generated excimers corresponding to green emission. Given the similar crystal packing and emission behavior of a bisanthracene linked by *meta*- phenylene,^3^ the spatially isolated dimeric anthracene moieties of 2a might promote excimer emission in a similar fashion, as disclosed by single crystal X-ray analysis. The other V-shaped bisanthracenes prepared here (2b–d and 3a) also showed excimer-like emission. The photoluminescence quantum yields of 2a–d and 3a were closer to each other in the solid state rather than in the solution state. Excimer formation may inhibit the non-radiative relaxation of 2a–d in solution state.

DFT calculations were performed for ADMS (Fig. S6†), BADMS (Fig. S7†), 2a (Fig. 4) and 3a (Fig. S8†). The optimized structures of 2a and 3a at the ωB97X-D/def2-SV(P) level showed good agreement with the crystal structures.^8^ The TD-DFT calculation at the ωB97X-D/def2-SVPD level suggested that the wavelengths at the absorption maxima of ADMS, BADMS and 2a should be 329 nm (*f* = 0.167), 344 nm (*f* = 0.222), and 345 nm (*f* = 0.328), respectively (Table S2†).^9^ The transitions of BADMS and 2a were derived from HOMO−1, HOMO, LUMO and LUMO+1 orbitals. Those frontier orbitals were delocalized between both of the anthracene moieties, meaning that there were some interactions of π-orbitals between them. In contrast, the diphenyldisilane moiety of 2a was contributed to the HOMO−2, and the intramolecular interaction between the diphenylsilane and the anthracene moieties was negligible for the absorption of visible light. Although the calculated transition energies of BADMS and 2a were very close, the experimental peaks were separated by 16 nm and the peak of BADMS was closer to that of ADMS than of 2a. The qualitative difference between the calculated and experimental peaks of BADMS may be explained by the high freedom of conformation in the experimental solution or by an overestimated π–π interaction in DFT calculation.

**Fig. 4:**
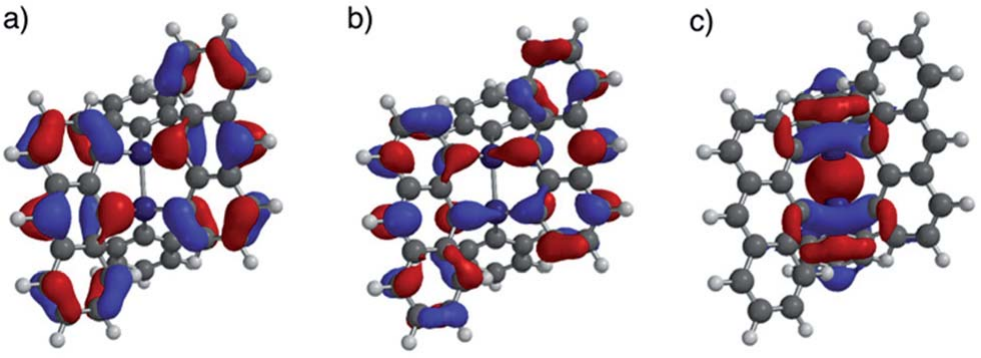
(a) HOMO, (b) LUMO, and (c) HOMO−2 lobes of 2a calculated at the ωB97X-D/def2-SVPD//ωB97X-D/def2-SV(P) level.

DFT optimization at the ωB97X-D/def2-SV(P) level also successfully reproduced the crystal structure of 3a. The Si–Si distance and Löwdin bond order were 2.760 Å and 0.17, respectively. A transition peak of 3a by TD-DFT calculation at the ωB97X-D/def2-SVPD appeared at 343 nm (*f* = 0.285) corresponding to HOMO–LUMO and HOMO−1–LUMO+1 transitions of the interacted anthracene moieties as seen in 2a. The calculated wavelength of 3a was 2 nm shorter than that of 2a, which quantitatively agreed with the experimental UV-vis absorption spectra.

## Conclusions

We demonstrated the dimerization of 9-anthrylarylsilanes catalyzed by RuH_2_(CO) (PPh_3_)_3_, affording V-shaped bisanthracenes with Si–Si single bonds. Contrary to the reported 7,8-disilabicyclo[3.3.0]octadienes that easily oxidized to siloxanes under air, the obtained V-shaped bisanthracenes were air-stable and oxidation proceeded smoothly in the presence of NMO. Single crystal X-ray analysis revealed intra- and intermolecular interactions between the anthracene moieties of the V-shaped bisanthracenes before and after oxidation. The intramolecular interaction probably induced a bathochromic shift in the chloroform solution of 2a as compared with 9-anthryldimethylsilane and bis(9-anthryl)dimethylsilane. Moreover, the intermolecular π-stacked dimers were spatially isolated from each other, leading to excimer emission in the solid state. It should be noted that Si–Si single bonds may influence fluorescence quantum yields in the solution state. Oxidation of 2a to 3a increased the quantum yield six-fold.

## Conflicts of interest

There are no conflicts to declare.

## Supplementary Material

RA-008-C8RA05293J-s001

RA-008-C8RA05293J-s002
