# Supplementary material for: Intra- and intermolecular interaction of anthracene moieties in 7,8-disilabicyclo[3.3.0]octadienyl-bridged bisanthracenes
Source: RSC Adv. 2018 Jul 13;8(44):25177–80. doi: 10.1039/c8ra05293j (PMC9082342; doi:10.1039/c8ra05293j)
Supplement: RA-008-C8RA05293J-s001 [file RA-008-C8RA05293J-s001.pdf]

Electronic Supplementary Material (ESI) for RSC Advances.
This journal is © The Royal Society of Chemistry 2018

Supporting Information

Intra- and Intermolecular Interaction of Anthracene Moieties in 7,8-Disilabicyclo[3.3.0]octadienyl-Bridged Bisanthracenes

Yuichiro Tokoro, * Nobuhiko Ohtsuka, Shin-ichi Fukuzawa, and Toshiyuki Oyama*

Table of Contents

1. General Information
2. Synthesis
3. Thermogravimetric Analysis
4. UV-vis and photoluminescence properties
5. DFT calculation
6. NMR spectra

Experimental Procedures

1. General Information

The ^{1}H, ^{13}C and ^{29}Si NMR spectra were recorded with a Bruker BioSpin DRX300 or DRX500 NMR (300 or 500 MHz) spectrometer. The chemical shifts are reported in δ units downfield from the internal reference (Me_4Si). Column chromatography was performed with silica gel (Fuji Silysia PSQ100B). High-resolution mass spectra (HRMS) were measured by a Hitachi High-Technologies Nano Frontier LD. UV/vis spectra were recorded on a SHIMADZU UV-1800 spectrophotometer. Photoluminescence spectra were recorded on a SHIMADZU RF-5300PC spectrofluorometer. Luminescence quantum yields were obtained by a JASCO FP-8200 spectrofluorometer. X-ray crystallographic analysis was carried out by a Rigaku XtaLAB PRO HPC diffractometer with Cu Kα radiation. The structures were solved and refined by Olex2. Thermogravimetric analysis (TGA) was carried out on Shimadzu TGA-50 at a heating rate of 10 ºC/min under a nitrogen flow. DFT calculation was performed with Spartan'16. Unless otherwise noted, available reagents were used without further purification. 4-Methoxyphenylsilane[1] were prepared as according to the literature.

2. Synthesis

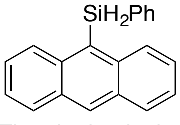

9-(Phenylsilyl)anthracene (1a). In an Ar purged J. Young tube, 9-bromoanthracene (1.54 g, 6.00 mmol) and tetrahydrofuran (24 mL) were placed and cooled to –78 ºC. Then, *n*BuLi (1.6 M in hexane, 4.0 mL, 6.4 mmol) was slowly added, followed by stirring for 30 min. After the addition of trichlorophenylsilane (1.06 mL, 6.60 mmol), the reaction mixture was stirred for 6 h at room temperature. The obtained mixture was added to lithium aluminium hydride (0.228 g, 6.00 mmol) in tetrahydrofuran (3 mL), followed by stirring for 24 h at room temperature. The reaction mixture was quenched with 1 M HCl aq, extracted with hexane, washed with sat. $NaHCO_3$ aq and brine, dried over Na_2SO_4, and concentrated. The residue was purified by recrystallization from ethanol to give a colorless solid (0.854 g, 3.00 mmol, 50 % yield). ^{1}H NMR (300 MHz, $CDCl_3$): δ = 8.62 (s, 1H, Ar-*H*), 8.55 (m, 2H, Ar-*H*), 8.08 (m, 2H, Ar-H), 7.61-7.48 (m, 6H, Ar-*H*), 7.40-7.26 (m, 3H, Ar-*H*), 5.70 (s, 2H, -Si*H*$_2$-) ppm. ^{13}C NMR (125 MHz, $CDCl_3$): δ = 137.8, 135.6, 131.7, 131.4, 131.1, 129.9, 129.5, 128.5, 128.3, 126.6, 126.3, 125.1 ppm. HRMS calcd for $C_{20}H_{16}Si$ $[M]^+$: 284.1016; found: 284.1020.

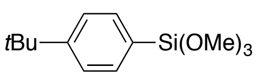

(4-*tert*-Butylphenyl)trimethoxysilane. Mg (2.04 g, 84.0 mmol) and LiCl (3.05 g, 72.0 mmol) were placed in a J. Young tube, which were dried by a heat gun under vacuum. After cooling to room temperature and purging Ar, THF (60 mL) and DIBAL-H (1.5 M in toluene, 0.6 mL, 0.9 mmol) were added. Then, 4-bromo-*tert*-butylbenzene (10.2 mL. 60.0 mmol) was slowly added and the reaction mixture was stirred for 4 h. The Grignard reagent was slowly added to tetramethyl orthosilicate (26.3 mL, 180 mmol) in THF (30 mL) at –45 ºC, followed by stirring for 12 h at room temperature. The mixture was diluted with hexane (180mL), filtered through a plug of Celite® and concentrated. The resulting liquid was purified via distillation (b.p. = 77 ºC, ~0.5 Torr) to give a colorless liquid (9.07 g, 35.7 mmol, 60% yield). ^{1}H NMR (500 MHz, $CDCl_3$): δ = 7.59 (d, 2H, Ar-*H*, *J* = 8.3 Hz), 7.42 (d, 2H, Ar-*H*, *J* = 8.3 Hz), 3.62 (s, 9H, -Si(OC*H*$_3$)$_3$), 1.32 (s, 9H, -C(C*H*$_3$)$_3$) ppm. ^{13}C NMR (125 MHz, $CDCl_3$): δ = 153.8, 134.8, 126.0, 125.1, 50.9, 34.9, 31.3 ppm. ^{29}Si NMR (99 MHz, $CDCl_3$): δ = –53.2 ppm.

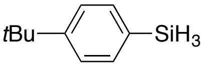

(4-*tert*-Butylphenyl)silane. To a 2-neck round bottom flask was added $LiAlH_4$ (2.58 g, 68.0 mmol) and Et_2O (68 mL). The mixture was cooled to –45 ºC and (4-*tert*-butylphenyl)trimethoxysilane was added slowly and allowed to stir for 12 h at room temperature. The excess $LiAlH_4$ was quenched by the addition of EtOAc (53 mL, 544 mmol) at –45 ºC. The mixture was filtered through a plug of Celite® and concentrated to give a colorless liquid (quantitative yield). ^{1}H NMR (500 MHz, $CDCl_3$): δ = 7.53 (d, 2H, Ar-*H*, *J* = 8.1 Hz), 7.40 (d, 2H, Ar-*H*, *J* = 8.1 Hz), 4.19 (s, 3H, -Si*H*$_3$), 1.32 (s, 9H, -C(C*H*$_3$)$_3$) ppm. ^{13}C NMR (125 MHz, $CDCl_3$): δ = 153.2, 135.9, 125.3, 124.8, 34.9, 31.3 ppm. ^{29}Si NMR (99 MHz, $CDCl_3$): δ = –59.2 ppm.

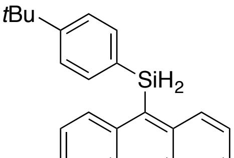

9-(4-*tert*-Butylphenylsilyl)anthracene (1b). To a J. Young tube was added (4-*tert*-butylphenyl)silane (1.36 g, 8.28 mmol) and hexane (12 mL) and cooled to 0 ºC. BCl_3 (1 M in CH_2Cl_2, 3.3 mL, 3.3 mmol) was added and the reaction mixture was stirred for 21 h at room temperature. All the volatiles were removed under reduced pressure and the residue was dissolved in hexane (3 mL), followed by addition to 9-anthryllithium (6.00 mmol, generated by treating 9-bromoanthracene (1.54 g, 6.00 mmol) with *n*-butyllithium (4.0 mL, 6.4 mmol, 1.6 M solution in hexane) in diethyl ether (24 mL) for 30 min at –45 ºC) solution at –45 ºC. After stirring for 1.5 h at room temperature, the reaction mixture was quenched with NH_4Cl aq, extracted with hexane, washed with water and brine, dried over Na_2SO_4, and concentrated. The residue was subjected to column chromatography on silica gel with hexane followed by recrystallization from CH_2Cl_2/MeOH to give a pale yellow solid (1.35 g, 3.96 mmol, 66% yield). ^{1}H NMR (500 MHz, $CDCl_3$): δ = 8.59 (s, 1H, Ar-*H*), 8.55 (m, 2H, Ar-*H*), 8.05 (m, 2H, Ar-*H*), 7.50 (m, 6H, Ar-*H*), 7.32 (d, 2H, Ar-*H*, *J* = 8.3 Hz), 5.66 (s, 2H, -Si*H*$_2$-), 1.26 (s, 9H, -C(C*H*$_3$)$_3$) ppm. ^{13}C NMR (125 MHz, $CDCl_3$): δ = 153.0, 137.8, 135.5, 131.4, 131.0, 129.5, 128.6, 127.9, 127.1, 126.2, 125.3, 125.1 ppm. ^{29}Si NMR (99 MHz, $CDCl_3$): δ = –49.3 ppm. HRMS calcd for $C_{24}H_{24}Si$ $[M]^+$: 340.1642, found: 340.1648.

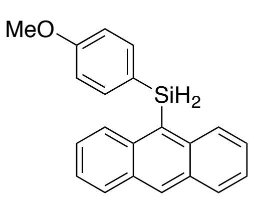

9-(3-Methoxyphenylsilyl)anthracene (1c). According to the procedure for **1b**, the reaction using 4-methoxyphenylsilane (0.820 g, 5.93 mmol), BCl_3 (1 M in CH_2Cl_2, 2.4 mL, 2.4 mmol), 9-bromoanthracene (1.11 g, 4.30 mmol) and *n*-butyllithium (2.9 mL, 4.6 mmol, 1.6 M solution in hexane) afforded 0.414 g (1.32 mmol, 22% yield) of a yellow solid. ^{1}H NMR (500 MHz, $CDCl_3$): δ = 8.56 (s, 1H, Ar-*H*), 8.52 (dd, 2H, Ar-*H*, *J* = 7.4, 2.3 Hz), 7.49-7.44 (m, 6H, Ar-*H*), 6.81 (d, 2H, Ar-*H*, *J* = 8.7 Hz), 5.65 (s, 2H, -SiH_2-), 3.72 (s, 3H, -OCH_3) ppm. ^{13}C NMR (125 MHz, $CDCl_3$): δ = 161.2, 137.8, 137.1, 131.4, 131.0, 129.5, 128.5, 127.2, 126.2, 125.1, 122.1, 114.2, 55.1 ppm. HRMS calcd for $C_{21}H_{18}OSi$ $[M]^+$: 314.1122, found: 314.1116.

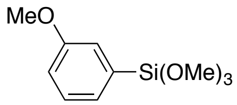

(3-Methoxyphenyl)trimethoxysilane. According to the procedure for (4-*tert*-butylphenyl)trimethoxysilane, the reaction using 3-bromoanisole (7.5 mL, 60 mmol), Mg (2.04 g, 84.0 mmol), LiCl (3.05 g, 72.0 mmol) and tetramethyl orthosilicate (26.3 mL, 180 mmol) afforded 5.61 g (24.6 mmol, 41% yield) of a colorless oil. ^{1}H NMR (500 MHz, $CDCl_3$): δ = 7.33 (ddd, 1H, Ar-*H*, *J* = 8.2, 7.2, 0.5 Hz), 7.23 (dt, 1H, Ar-*H*, *J* = 7.2, 1.0 Hz), 7.18 (d, Ar-*H*, 1H, *J* = 2.8 Hz), 6.99, (ddd, 1H, Ar-*H*, *J* = 8.3, 2.8, 1.1 Hz), 3.83 (s, 3H, Ar-OCH_3), 3.63 (s, 9H, Si-OCH_3) ppm. ^{13}C NMR (125 MHz, $CDCl_3$): δ = 159.3, 131.0, 129.4, 127.1, 119.8, 116.7, 55.3, 51.0 ppm. ^{29}Si NMR (99 MHz, $CDCl_3$): δ = –54.1 ppm. HRMS calcd for $C_{10}H_{17}O_4Si$ $[M+H]^+$: 229.0891, found: 229.0891.

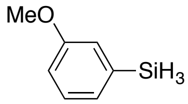

(3-Methoxyphenyl)silane. According to the procedure for (4-*tert*-butylphenyl)silane, the reaction using (3-methoxyphenyl)trimethoxysilane (5.48 g, 24.0 mmol) and $LiAlH_4$ (1.82 g, 48.0 mmol) afforded 3.09 g (22.4 mmol, 93% yield) of a colorless oil. Yield: 93%. ^{1}H NMR (500 MHz, $CDCl_3$): δ = 7.30 (dd, 1H, Ar-*H*, *J* = 8.2, 7.2 Hz), 7.17 (dt, 1H, Ar-*H*, *J* = 7.2, 1.0 Hz), 7.12 (dd, Ar-*H*, 1H, *J* = 2.7, 0.6 Hz), 6.95, (ddd, 1H, Ar-*H*, *J* = 8.3, 2.7, 0.9 Hz), 4.19 (s, 3H, -SiH_3), 3.82 (s, 3H, Ar-OCH_3) ppm. ^{13}C NMR (125 MHz, $CDCl_3$): δ = 159.2, 129.8, 129.5, 128.2, 120.9, 115.8, 55.3 ppm. ^{29}Si NMR (99 MHz, $CDCl_3$): δ = –58.0 ppm.

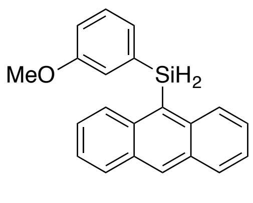

9-(3-Methoxyphenylsilyl)anthracene (1d). According to the procedure for **1b**, the reaction using 3-methoxyphenylsilane (1.14 g, 8.28 mmol), BCl_3 (1 M in CH_2Cl_2, 3.3 mL, 3.3 mmol), 9-bromoanthracene (1.54 g, 6.00 mmol) and *n*-butyllithium (4.0 mL, 6.4 mmol, 1.6 M solution in hexane) afforded 1.10 g (3.50 mmol, 58% yield) of a yellow solid. ^{1}H NMR (500 MHz, $CDCl_3$): δ = 8.59 (s, 1H, Ar-*H*), 8.52 (d, 2H, Ar-*H*, *J* = 9.4 Hz), 8.05 (m, 2H, Ar-*H*), 7.52-7.46 (m, 4H, Ar-*H*), 7.22 (t, 1H, Ar-*H*, *J* = 7.7 Hz), 7.12 (m, 2H, Ar-*H*), 6.89 (m, 1H, Ar-*H*), 5.66 (s, 2H, -SiH_2-), 3.70 (s, 3H, -OCH_3) ppm. ^{13}C NMR (125 MHz, $CDCl_3$): δ = 159.3, 137.9, 133.2, 131.4, 131.1, 129.54, 129.52, 128.5, 127.8, 126.5, 126.3, 125.1, 120.8, 115.3, 55.2 ppm. ^{29}Si NMR (99 MHz, $CDCl_3$): δ = –48.5 ppm. HRMS calcd for $C_{21}H_{18}OSi$ $[M]^+$: 314.1122, found: 314.1127.

General procedure for ruthenium-catalyzed dimerization of 9-anthrylarylsilanes. In an Ar purged J. Young tube, cyclopentyl methyl ether (1.0 mL), $[RuH_2(CO)(PPh_3)_3]$ (18.4 mg, 0.020 mmol), 9-anthrylarylsilane (0.40 mmol) and cyclooctene (0.80 mmol) were placed. After stirring for 16 h at 115 °C, the precipitate was collected by filtration at room temperature.

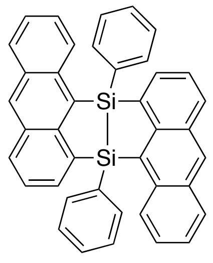

2a. According to the general procedure, the reaction of **1a** (114 mg, 0.400 mmol) afforded 43.8 mg (77.8 μmol, 39% yield) of a yellow solid. ^{1}H NMR (300 MHz, $CDCl_3$): δ = 8.50 (s, 2H, Ar-*H*), 8.37 (m, 4H, Ar-*H*), 8.03 (m, 4H, Ar-*H*), 7.69 (m, 4H, Ar-*H*), 7.56 (dd, 2H, Ar-*H*, *J* = 8.4, 6.5 Hz), 7.46-7.28 (m, 10H, Ar-*H*) ppm. ^{13}C NMR (125 MHz, $CDCl_3$): δ = 138.9, 137.2, 136.5, 136.4, 134.9, 132.0, 131.7, 131.3, 130.71, 130.66, 130.5, 129.7, 129.3, 128.4, 125.9, 125.3 ppm. HRMS (ESI-TOF) calcd for $C_{40}H_{26}Si_2$: 562.1573, found: 562.1545. T_{d5} = 401 °C.

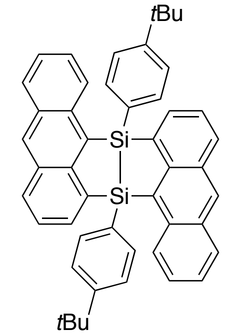

2b. According to the general procedure, the reaction of **1b** (136 mg, 0.400 mmol) afforded 37.0 mg (54.8 μmol, 27% yield) of a yellow solid. ^{1}H NMR (500 MHz, $CDCl_3$): δ = 8.48 (s, 2H, Ar-*H*), 8.38 (m, 2H, Ar-*H*), 8.36 (dd, 2H, Ar-*H*, *J* = 6.5, 1.3 Hz), 8.02 (ddd, 2H, Ar-*H*, *J* = 8.5, 1.3, 0.6 Hz), 7.99 (m, 2H, Ar-*H*), 7.63 (d, 4H, Ar-*H*, *J* = 8.5 Hz), 7.54 (dd, 2H, Ar-*H*, *J* = 8.4, 6.4 Hz), 7.41 (m, 4H, Ar-*H*), 7.34 (d, 4H, Ar-*H*, *J* = 8.5 Hz), 1.30 (s, 18H, -C(CH_3)$_3$) ppm. ^{13}C NMR (125 MHz, $CDCl_3$): δ = 152.7, 146.7, 139.4, 137.1, 136.8, 136.6, 134.7, 131.9, 131.7, 130.8, 130.5, 130.4, 129.3, 127.6, 125.6, 125.4, 125.22, 125.17, 34.9, 31.3 ppm. ^{29}Si NMR (99 MHz, $CDCl_3$): δ = -27.2 ppm.

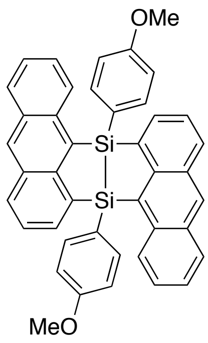

2c. According to the general procedure, the reaction of **1c** (126 mg, 0.400 mmol) afforded 36.9 mg (59.2 μmol, 30% yield) of a yellow solid. ^{1}H NMR (500 MHz, $CDCl_3$): δ = 8.46 (s, 2H, Ar-*H*), 8.41 (m, 2H, Ar-*H*), 8.33 (dd, 2H, Ar-*H*, *J* = 6.4, 1.2 Hz), 7.99 (m, 4H, Ar-*H*), 7.60 (d, 4H, Ar-*H*, *J* = 8.8 Hz), 7.53 (dd, 2H, Ar-*H*, *J* = 8.4, 6.4 Hz), 7.41 (m, 4H, Ar-*H*), 6.86 (d, 4H, Ar-*H*, *J* = 8.8 Hz), 3.77 (s, 6H, -OC*H*$_3$) ppm. ^{13}C NMR (125 MHz, $CDCl_3$): δ = 161.1, 146.8, 139.4, 138.0, 137.2, 136.8, 134.7, 131.9, 131.7, 130.7, 130.5, 130.4, 129.3, 125.7, 125.3, 125.2, 121.5, 114.4, 55.2 ppm. HRMS (ESI-TOF) calcd for $C_{42}H_{31}O_2Si_2$ $[M+H]^+$: 623.1857, found: 623.1875.

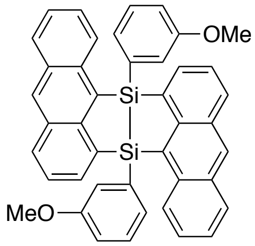

2d. According to the general procedure, the reaction of **1d** (126 mg, 0.400 mmol) afforded 55.5 mg (89.1 μmol, 45% yield) of a yellow solid. ^{1}H NMR (500 MHz, CD_2Cl_2): δ = 8.54 (s, 2H, Ar-*H*), 8.39 (m, 4H, Ar-*H*), 8.04 (m, 4H, Ar-*H*), 7.58 (dd, 2H, Ar-*H*, *J* = 8.4, 6.5 Hz), 7.46 (m, 4H, Ar-*H*), 7.26 (m, 6H, Ar-*H*), 6.94 (m, 2H, Ar-*H*), 3.64 (s, 6H, -OC*H*$_3$) ppm. ^{13}C NMR (125 MHz, CD_2Cl_2): δ = 159.9, 147.0, 138.8, 137.3, 136.4, 135.3, 132.9, 132.2, 132.0, 131.0, 130.8, 130.7, 129.9, 129.6, 128.8, 126.2, 125.7, 125.6, 121.8, 115.6, 55.4 ppm. HRMS (ESI-TOF) calcd for $C_{42}H_{30}O_2Si_2$ $[M]^+$: 622.1779, found: 622.1783.

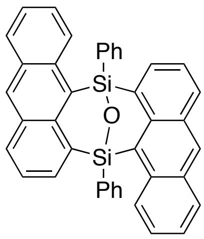

3a. In an Ar purged J. Young tube, toluene (0.8 mL), **2a** (22,5 mg, 0.040 mmol) and NMO (9.4 mg, 0.080 mmol) were placed. After stirring for 9 h at 80 ºC, ethanol was added to precipitate the products. The precipitate was collected by filtration, affording 21.8 mg (37.7 μmol, 94% yield) as a yellow solid. ^{1}H NMR (500 MHz, CD_2Cl_2): δ = 8.58 (s, 2H, Ar-*H*), 8.30 (dd, 2H, Ar-*H*, *J* = 6.4, 1.4 Hz), 8.27 (dd, 2H, Ar-*H*, *J* = 8.8, 0.8 Hz), 8.13 (ddd, 2H, Ar-H, *J* = 8.5, 1.4, 0.6 Hz), 7.98 (dt, 2H, Ar-*H*, *J* = 8.4, 0.6 Hz), 7.78 (dd, 4H, Ar-*H*, *J* = 8.1, 1.4 Hz), 7.59 (dd, 2H, Ar-*H*, *J* = 8.5, 6.4 Hz), 7.51 (ddt, 2H, Ar-*H*, *J* = 8.1, 6.9, 1.3 Hz), 7.42-7.38 (m, 6H, Ar-*H*), 7.29 (ddd, 2H, Ar-*H*, *J* = 8.8, 6.6, 1.4 Hz) ppm. ^{13}C NMR (125 MHz, $CDCl_3$): δ = 142.9, 136.9, 136.3, 134.2, 133.9, 133.3, 132.7, 132.4, 132.2, 131.3, 131.2, 129.8, 129.7, 129.5, 128.6, 125.6, 125.4, 124.6 ppm. HRMS (ESI-TOF) calcd for $C_{40}H_{27}OSi_2$ $[M+H]^+$: 579.1595, found: 579.1595.

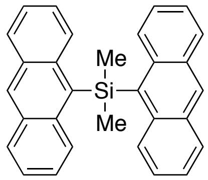

Bis(9-anthryl)dimethylsilane (BADMS). In an Ar purged J. Young tube, 9-bromoanthracene (1.54 g, 6.00 mmol) and diethyl ether (30 mL) were placed and cooled to –45 ºC. Then, *n*BuLi (1.6 M in hexane, 4.0 mL, 6.4 mmol) was slowly added, followed by stirring for 30 min. After the addition of dichlorodimethylsilane (0.36 mL, 3.00 mmol), the reaction mixture was stirred for 49 h at room temperature. The mixture was quenched with NH_4Cl aq, extracted with toluene, washed with water and brine, dried over Na_2SO_4, and concentrated. The residue was purified by recrystallization from dichloromethane/ethanol to give 0.724 g (1.75 mmol, 50 % yield) of a colorless solid. ^{1}H NMR (500 MHz, $CDCl_3$): δ = 8.52 (dd, 4H, Ar-*H*, *J* = 9.0, 0.8 Hz), 8.46 (s, 2H, Ar-*H*), 7.97 (dt, 4H, Ar-H, *J* = 8.4, 0.7 Hz), 7.34 (ddd, 4H, Ar-*H*, *J* = 8.4, 6.5, 1.0 Hz), 7.19 (ddd, 4H, Ar-*H*, *J* = 9.0, 6.5, 1.4 Hz), 1.25 (s, 6H, -C*H*$_3$) ppm. ^{13}C NMR (125 MHz, $CDCl_3$): δ = 137.27, 136.32, 131.58, 130.27, 129.72, 128.21, 125.4, 124.7, 8.2 ppm. ^{29}Si NMR (99 MHz, $CDCl_3$): δ = -9.1 ppm.

3. Thermogravimetric Analysis

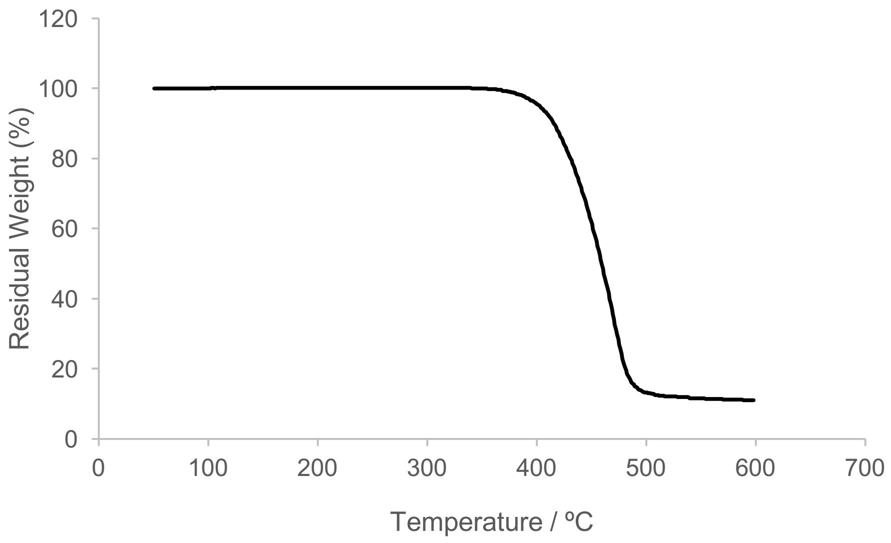

Figure S1. TGA thermogram of **2a** (heating rate: 10 ºC/min, under N_2 (50 mL/min)).

4. UV-vis and Photoluminescence Properties

Table S1. Photophysical properties of ADMS, BADMS, **2** and **3a**.

Compound	λ_{abs}/nm[a],[b] (ε/10^{-5} $M^{-1}cm^{-1}$)[c]	$\lambda_{em,chloroform}$/nm[a],[d]	$\Phi_{f,chloroform}$[a],[e]	$\lambda_{em,powder}$/nm[d]	$\Phi_{f,powder}$[e]
ADMS	351 (0.055), 369 (0.083), 390 (0.077)	396, 419	0.50	427, 447	0.46
BADMS	355 (0.143), 374 (0.234), 395 (0.259)	406, 424	0.40	435	0.02
**2a**	368 (0.159), 388 (0.226), 412 (0.295)	422, 445	0.10	560	0.10
**2b**	368 (0.111), 389 (0.154), 413 (0.200)	424, 444	0.06	536	0.12
**2c**	367 (0.108), 388 (0.142), 413 (0.178)	427, 441	0.02	541	0.13
**2d**	368 (0.096), 389 (0.136), 412 (0.178)	423, 444	0.17	532	0.25
**3a**	367 (0.117), 386 (0.160), 409 (0.177)	420, 443	0.60	470	0.15

[a] 10 μM in chloroform. [b] Observed absorption maximum at the longest wavelength. [c] Molar extinction coefficient. [d] Observed fluorescent maxima. [e] Fluorescent quantum yield determined by a calibrated integrating sphere system.

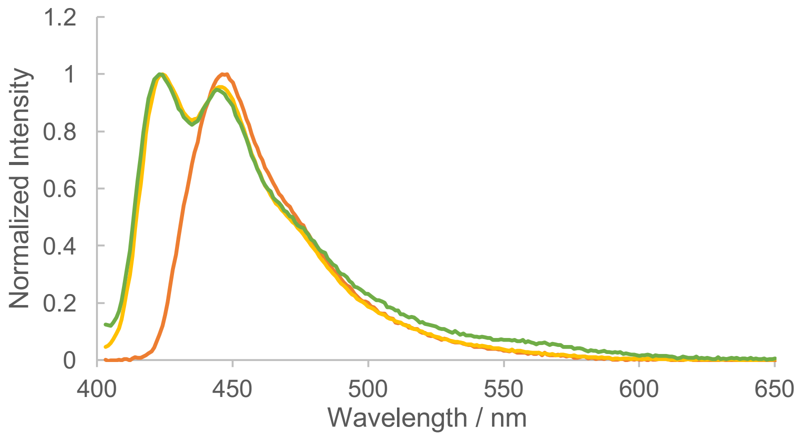

Figure S2. Normalized photoluminescence spectra of **2a** solutions (1.0 mM (red), 0.10 μM (yellow) and 0.10 μM (green) in chloroform).

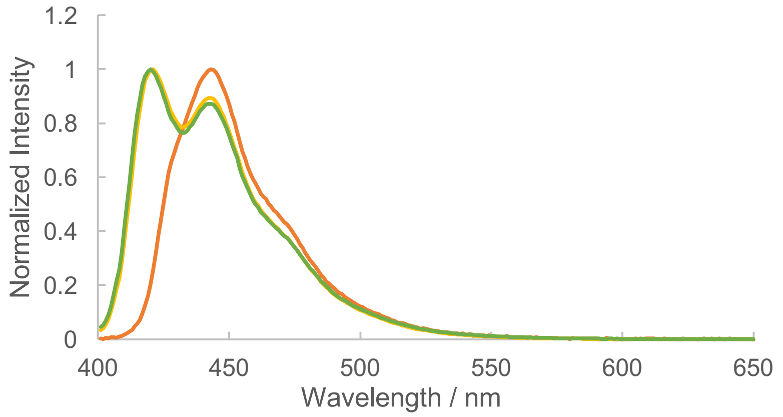

Figure S3. Normalized photoluminescence spectra of **3a** solutions (1.0 mM (red), 0.10 μM (yellow) and 0.10 μM (green) in chloroform).

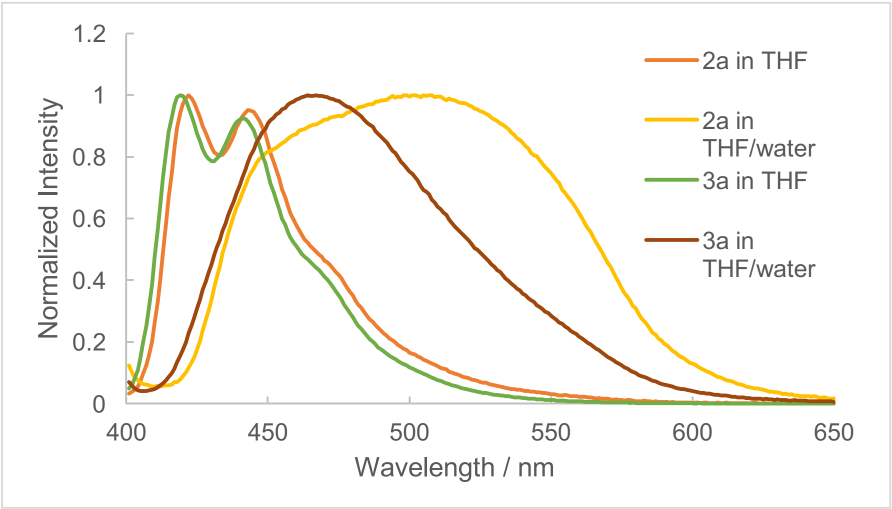

Figure S4. Normalized photoluminescence spectra of **2a** in THF (10 μM), **2a** in THF/water (1:99 (v/v), 10 μM), **3a** in THF (10 μM) and **3a** in THF/water (1:99 (v/v), 10 μM).

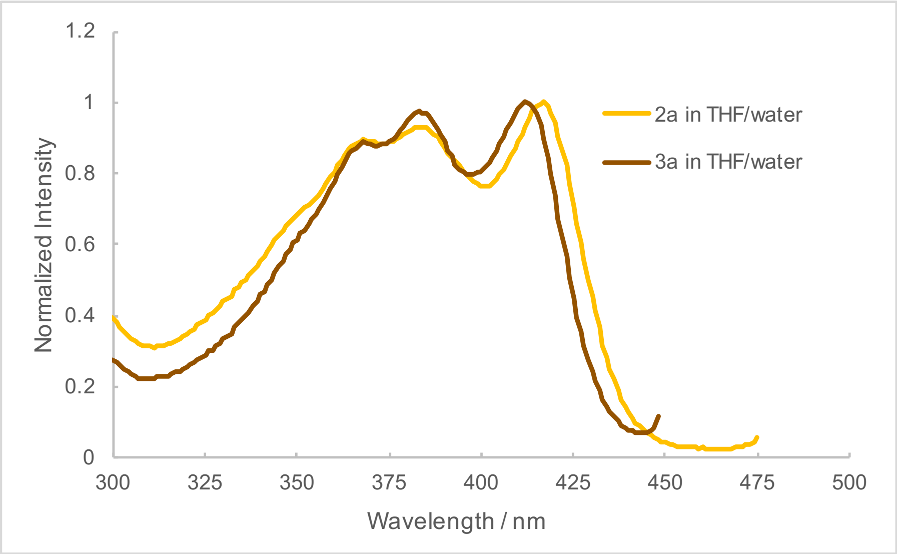

Figure S5. Normalized excitation spectra of **2a** in THF/water (1:99 (v/v), 10 μM, Em: 490 nm) and **3a** in THF/water (1:99 (v/v), 10 μM, Em: 463 nm).

5. DFT calculation

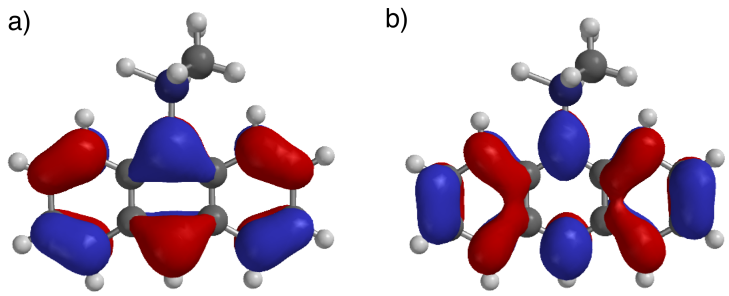

Figure S6. a) HOMO (–7.3 eV) and b) LUMO (–0.4 eV) lobes of ADMS calculated at ωB97X-D/def2-SVPD//ωB97X-D/def2-SV(P) level.

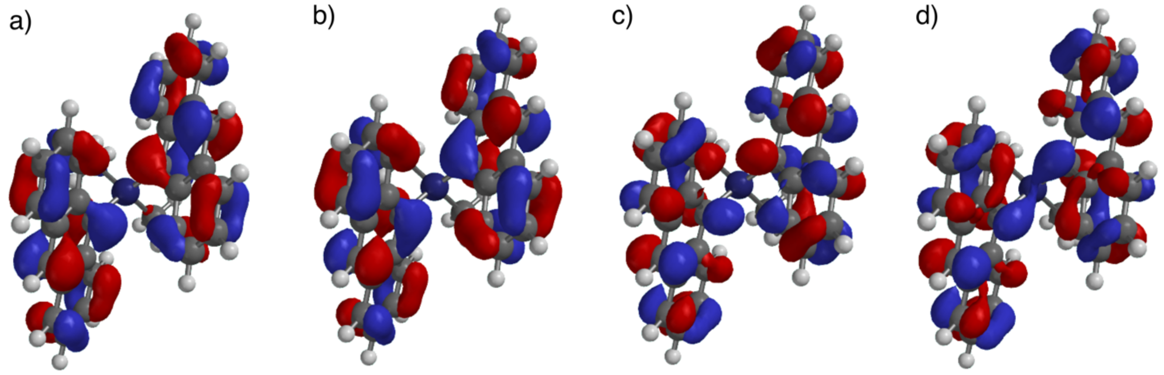

Figure S7. a) HOMO–1 (–7.3 eV), b) HOMO (–7.2 eV), c) LUMO (–0.4 eV) and d) LUMO+1 (–0.3 eV) lobes of BADMS calculated at ωB97X-D/def2-SVPD//ωB97X-D/def2-SV(P) level.

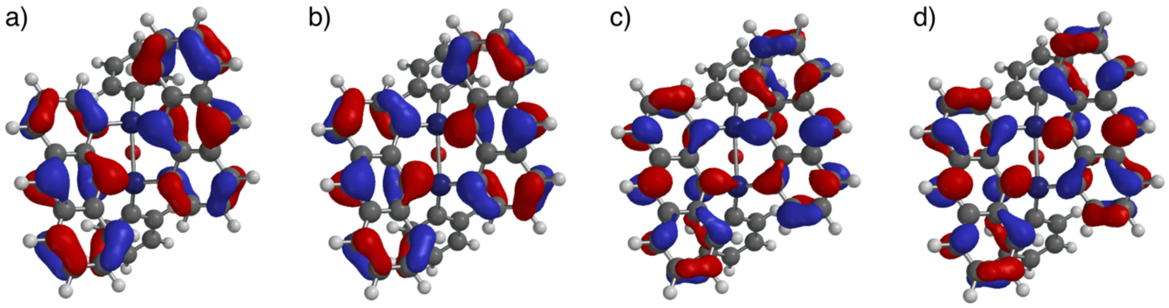

Figure S8. a) HOMO–1 (–7.4 eV), b) HOMO (–7.3 eV), c) LUMO (–0.6 eV) and d) LUMO+1 (–0.4 eV) lobes of **3a** calculated at ωB97X-D/def2-SVPD//ωB97X-D/def2-SV(P) level.

Table S2. Results of TD-DFT Calculation at ωB97X-D/def2-SVPD//ωB97X-D/def2-SV(P) level.

Compound	Energy gap / nm	Oscillator strength	MO component
ADMS	329	0.167	HOMO-LUMO (92%)
BADMS	327	0.115	HOMO–1-LUMO (46%)<br>HOMO-LUMO+1 (45%)
	344	0.222	HOMO-LUMO (69%)<br>HOMO–1-LUMO+1 (25%)
**2a**	330	0.146	HOMO–1-LUMO (53%)<br>HOMO-LUMO+1 (37%)
	345	0.328	HOMO-LUMO (74%)<br>HOMO–1-LUMO+1 (19%)
**3a**	331	0.118	HOMO–1-LUMO (54%)<br>HOMO-LUMO+1 (37%)
	343	0.285	HOMO-LUMO (68%)<br>HOMO–1-LUMO+1 (25%)

Table S3. Cartesian coordinates of ADMS optimized by DFT calculation at ωB97X-D/def2-SV(P) level.

Atom	X	Y	Z
H	-0.495031	5.366225	0.232877
C	-0.394413	4.2775	0.25685
H	-0.367187	4.063238	-1.872124
C	-0.323913	3.560623	-0.900961
C	-0.210628	2.235832	1.560483
C	-0.191843	2.133291	-0.883268
C	-0.335702	3.595692	1.506261
C	-0.132188	1.42662	0.372617
C	-0.119455	1.420645	-2.079309
H	-0.391365	4.169341	2.435975
H	-0.169476	1.755692	2.536735
C	0.00845	0.032236	-2.094344
H	-0.163957	1.96425	-3.028788
C	0.082444	-0.679854	-3.335814
C	0.066127	-0.683213	-0.846984
H	0.240945	-2.711612	-0.029636
C	-0.002453	0.011244	0.390955
C	0.206518	-2.038047	-3.364723
H	0.037284	-0.104151	-4.265455
H	0.262658	-2.572279	-4.317152
C	0.262263	-2.762857	-2.139587
H	0.360067	-3.85205	-2.160716
C	0.194898	-2.112863	-0.939266
Si	0.085623	-0.995352	2.010411

H	0.013381	-0.08588	3.192788
C	-1.394356	-2.153926	2.18301
H	-2.329133	-1.565285	2.175274
H	-1.340737	-2.687363	3.149187
H	-1.470748	-2.907377	1.381675
C	1.73775	-1.892714	2.179082
H	1.787438	-2.397465	3.160779
H	2.564496	-1.161462	2.13501
H	1.922246	-2.648679	1.39816

Table S4. Cartesian coordinates of BADMS optimized by DFT calculation at ωB97X-D/def2-SV(P) level.

Atom	X	Y	Z
Si	0	0	-1.683349
C	-0.249529	-1.492037	-2.820559
H	-0.706582	-2.363038	-2.321551
H	0.76043	-1.797648	-3.150783
H	-0.833762	-1.258916	-3.727569
C	0.249529	1.492037	-2.820559
H	0.706582	2.363038	-2.321551
H	-0.76043	1.797648	-3.150783
H	0.833762	1.258916	-3.727569
H	1.381986	3.201527	1.978503
C	0.404248	2.720398	1.886533
H	0.889264	1.893987	0.008181
C	0.122659	1.972913	0.778438
C	-1.794058	2.312743	2.785457
C	-1.146354	1.320565	0.597937
C	-0.56358	2.88606	2.918895
C	-2.126609	1.532034	1.630366
C	-1.448024	0.50587	-0.525979
H	-0.319763	3.480625	3.803816
H	-4.132479	1.112187	2.293317
H	-2.557338	2.443317	3.558901
C	-2.774675	0.027939	-0.6769
C	-3.22647	-0.681439	-1.841935
C	-3.752622	0.246965	0.35783
H	-5.79494	-0.103326	1.020794
C	-3.395086	0.968105	1.496739
C	-4.503789	-1.154743	-1.956795

H	-2.543474	-0.821337	-2.676924
H	-4.807287	-1.678209	-2.868017
C	-5.448534	-0.963568	-0.907878
H	-6.464609	-1.354609	-1.010765
C	-5.078643	-0.276654	0.211622
H	4.807287	1.678209	-2.868017
C	4.503789	1.154743	-1.956795
H	2.543474	0.821337	-2.676924
C	3.22647	0.681439	-1.841935
C	5.078643	0.276654	0.211622
C	2.774675	-0.027939	-0.6769
C	5.448534	0.963568	-0.907878
C	3.752622	-0.246965	0.35783
C	1.448024	-0.50587	-0.525979
H	6.464609	1.354609	-1.010765
H	4.132479	-1.112187	2.293317
H	5.79494	0.103326	1.020794
C	1.146354	-1.320565	0.597937
C	-0.122659	-1.972913	0.778438
C	2.126609	-1.532034	1.630366
H	2.557338	-2.443317	3.558901
C	3.395086	-0.968105	1.496738
C	-0.404248	-2.720398	1.886533
H	-0.889264	-1.893987	0.008182
H	-1.381986	-3.201527	1.978503
C	0.56358	-2.88606	2.918895
H	0.319763	-3.480625	3.803816
C	1.794058	-2.312743	2.785457

Table S5 Cartesian coordinates of **2a** optimized by DFT calculation at ωB97X-D/def2-SV(P) level.

Atom	X	Y	Z
H	-1.697513	-6.572949	-2.634768
C	-1.309429	-5.631451	-2.235779
H	0.617596	-5.857318	-3.140998
C	-0.031918	-5.237783	-2.514716
C	-1.680002	-3.631919	-0.913238
C	0.489082	-4.006321	-2.000017
C	-2.14585	-4.811318	-1.42283

C	-0.34413	-3.176636	-1.17249
C	1.793269	-3.592783	-2.286213
H	-3.169097	-5.131329	-1.20698
H	-2.337917	-3.022255	-0.289115
C	2.303822	-2.387009	-1.800535
H	2.429813	-4.229121	-2.910317
C	3.64226	-1.972896	-2.101141
C	1.47447	-1.548276	-0.977723
C	0.155981	-1.961645	-0.653499
C	4.136815	-0.794972	-1.621201
H	4.262015	-2.624833	-2.72486
H	5.160777	-0.488845	-1.853452
C	3.31323	0.050763	-0.820517
H	3.736051	0.994146	-0.45736
C	2.021381	-0.288096	-0.504284
Si	0.826646	0.794285	0.475992
Si	-0.827948	-0.792803	0.475022
H	-3.735606	-0.993567	-0.463741
C	-3.312435	-0.049923	-0.825794
C	-2.021104	0.289082	-0.507648
C	-3.639784	1.973838	-2.106777
C	-1.473479	1.549129	-0.980603
C	-4.134925	0.79586	-1.627545
C	-2.301653	2.38784	-1.804611
C	-0.155258	1.962256	-0.655005
H	-5.158582	0.489754	-1.861181
H	-2.425944	4.229822	-2.914805
H	-4.25888	2.625981	-2.730934
C	0.346228	3.176325	-1.174748
C	1.682559	3.630306	-0.915532
C	-0.485757	4.00596	-2.003528
H	-0.611999	5.856302	-3.145824
C	-1.790089	3.593212	-2.290276
C	2.149998	4.808503	-1.426419
H	2.340031	3.019863	-0.291648
H	3.173831	5.127123	-1.211307
C	1.314642	5.628891	-2.240204
H	1.703852	6.569644	-2.639847
C	0.036767	5.236396	-2.519136

C	1.627528	1.639651	1.944037
C	2.79035	2.862012	4.195118
C	2.725228	1.053956	2.594138
C	1.123443	2.850157	2.445977
C	1.698815	3.457265	3.561226
C	3.303239	1.658886	3.710148
H	3.139265	0.110584	2.222007
H	0.273235	3.333184	1.951938
H	1.294844	4.402169	3.936558
H	4.158716	1.188264	4.203762
H	3.243714	3.338844	5.069281
C	-1.631324	-1.63815	1.94173
C	-2.797188	-2.861647	4.190623
C	-1.126996	-2.848181	2.444613
C	-2.730809	-1.053538	2.589697
C	-3.310425	-1.659122	3.704562
C	-1.703791	-3.455766	3.558881
H	-0.275667	-3.330525	1.951833
H	-3.145109	-0.110618	2.216715
H	-4.167503	-1.189492	4.196346
H	-1.299564	-4.400212	3.935081
H	-3.251818	-3.338951	5.063867

Table S6. Cartesian coordinates of **3a** optimized by DFT calculation at ωB97X-D/def2-SV(P) level.

Atom	X	Y	Z
H	0.800644	-6.142389	-3.344309
C	0.826321	-5.214383	-2.766377
H	2.794757	-4.671564	-3.41086
C	1.925037	-4.40542	-2.802221
C	-0.286513	-3.687244	-1.243737
C	1.965404	-3.183218	-2.056202
C	-0.300641	-4.838922	-1.979152
C	0.848732	-2.806346	-1.234754
C	3.077883	-2.343379	-2.109701
H	-1.188659	-5.477173	-1.968472
H	-1.175983	-3.42903	-0.666215
C	3.102803	-1.119294	-1.441645
H	3.943399	-2.639916	-2.711551

C	4.242672	-0.259874	-1.571261
C	1.980306	-0.716805	-0.631327
C	0.883046	-1.610376	-0.47134
C	4.270631	0.963914	-0.974469
H	5.087287	-0.607744	-2.174133
H	5.140787	1.617827	-1.077821
C	3.136549	1.404076	-0.233087
H	3.164258	2.411733	0.197045
C	2.02271	0.620616	-0.055758
Si	-0.493576	-1.288632	0.80468
Si	0.494974	1.288304	0.805204
H	-3.163598	-2.410856	0.197191
C	-3.135541	-1.403215	-0.232982
C	-2.021226	-0.6203	-0.056137
C	-4.241406	0.261378	-1.570667
C	-1.978622	0.71718	-0.631429
C	-4.269707	-0.962449	-0.973957
C	-3.101175	1.120361	-1.441311
C	-0.88106	1.610232	-0.47142
H	-5.140079	-1.616062	-1.077307
H	-3.941364	2.641538	-2.710855
H	-5.085953	0.609483	-2.17348
C	-0.846273	2.806185	-1.23484
C	0.289705	3.686	-1.244195
C	-1.962883	3.183785	-2.056068
H	-2.791305	4.672729	-3.410706
C	-3.075887	2.344563	-2.109161
C	0.304696	4.837618	-1.979613
H	1.179444	3.426514	-0.667648
H	1.193265	5.475111	-1.969372
C	-0.822233	5.213927	-2.766455
H	-0.795943	6.14184	-3.344519
C	-1.921756	4.40598	-2.802062
O	0.000692	-0.000363	1.740703
C	0.834945	2.740494	1.932332
C	1.331542	4.817897	3.764015
C	1.967397	2.714431	2.762928
C	-0.048057	3.824849	2.04743
C	0.196264	4.85542	2.95423

C	2.216893	3.743985	3.669773
H	2.666294	1.873012	2.706721
H	-0.942741	3.869912	1.418331
H	-0.503793	5.692607	3.028981
H	3.104851	3.706698	4.307767
H	1.52523	5.627132	4.474529
C	-0.836653	-2.740773	1.930936
C	-1.338814	-4.818598	3.760422
C	0.040982	-3.830049	2.041517
C	-1.966542	-2.709994	2.764851
C	-2.218647	-3.739664	3.670814
C	-0.206245	-4.860995	2.94708
H	0.933339	-3.878663	1.409229
H	-2.660978	-1.864663	2.712086
H	-3.104294	-3.698614	4.311774
H	0.48919	-5.702354	3.017888
H	-1.534784	-5.628007	4.470116

6. NMR spectra

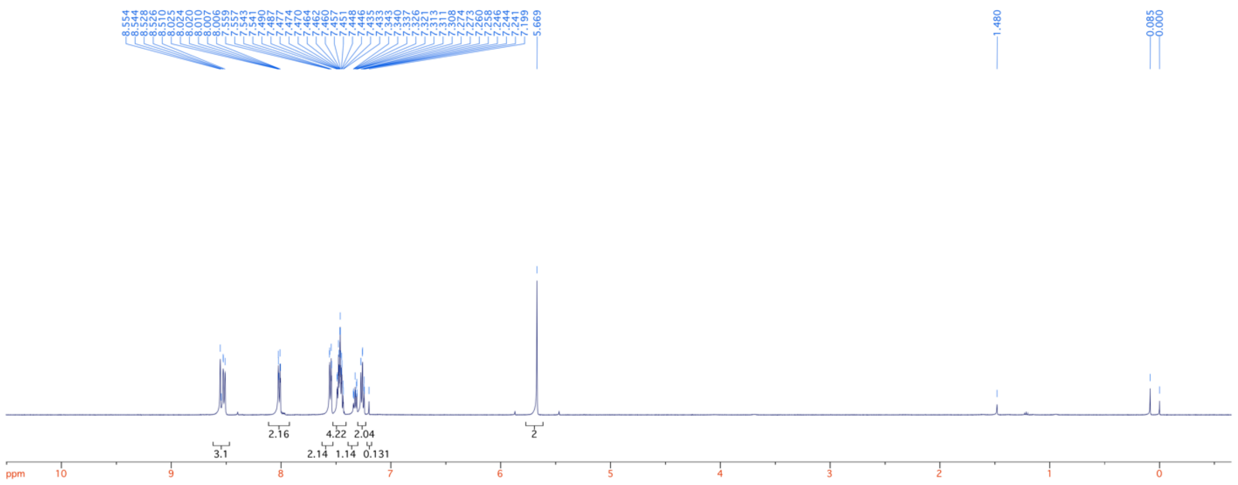

Figure S9. ^{1}H NMR spectrum of **1a**.

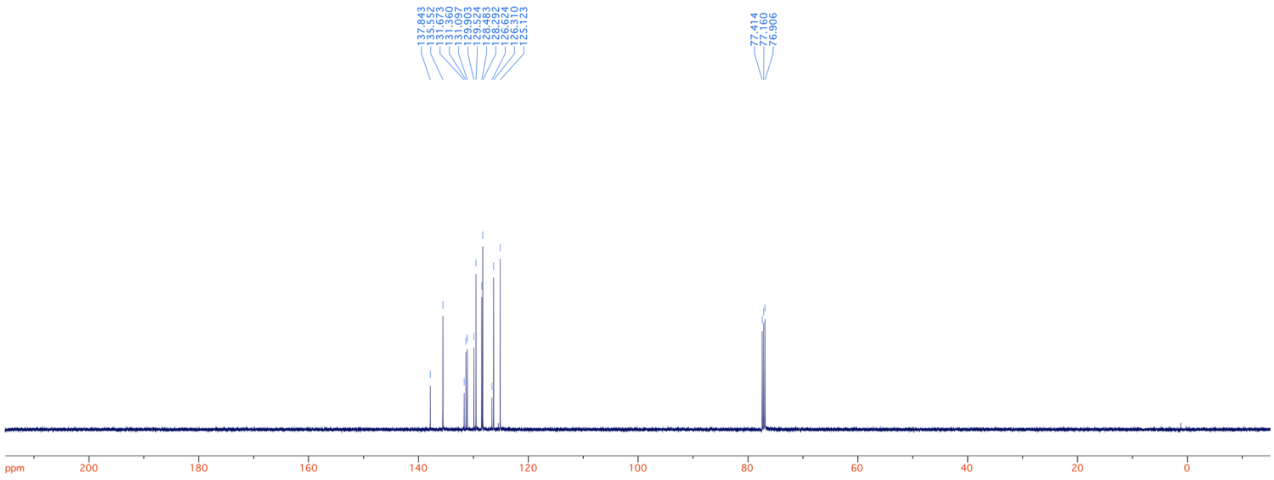

Figure S10. ^{13}C NMR spectrum of **1a**.

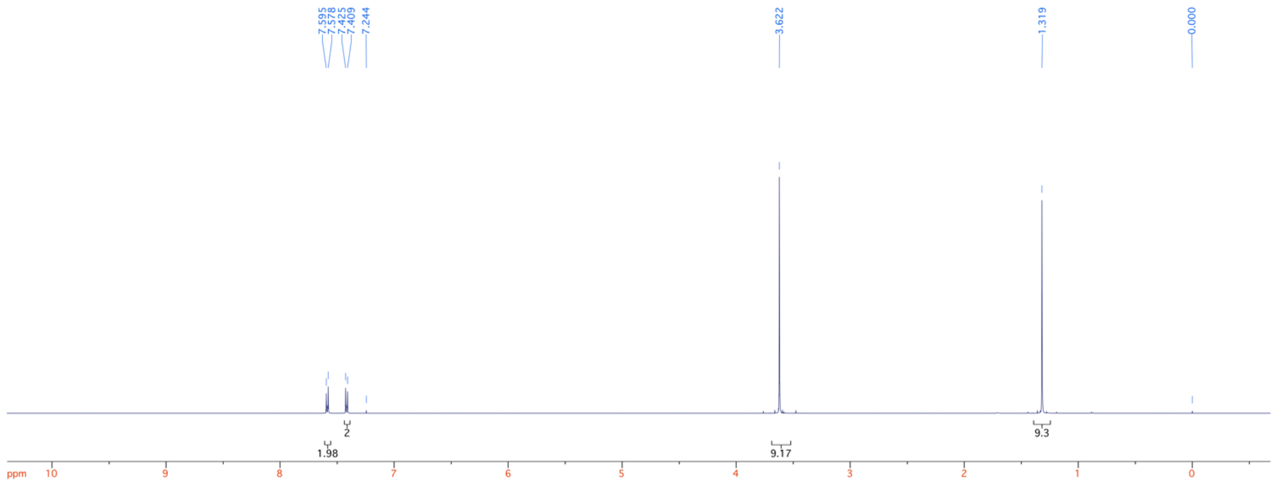

Figure S11. ^{1}H NMR spectrum of (4-*tert*-butylphenyl)trimethoxysilane.

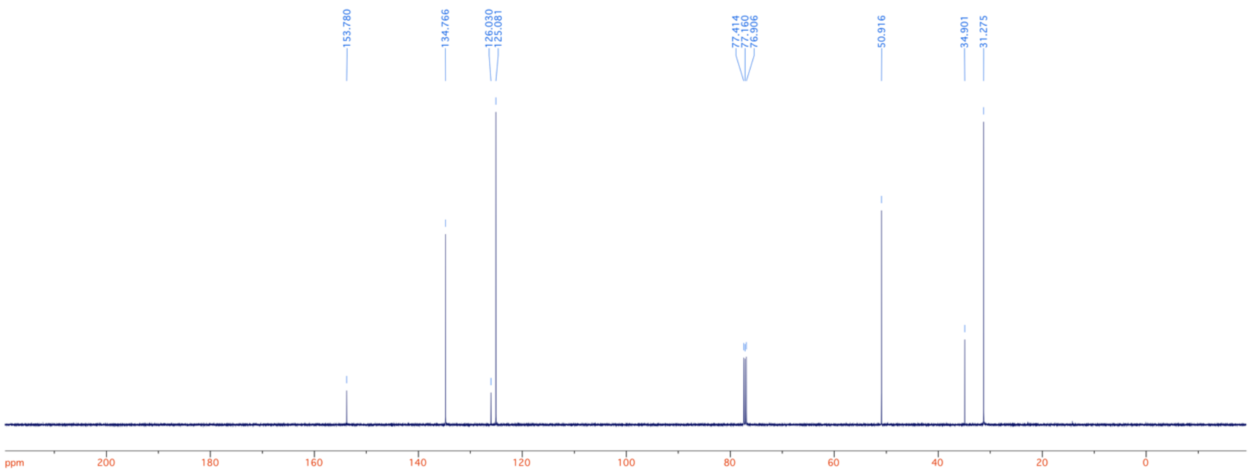

Figure S12. ^{13}C NMR spectrum of (4-*tert*-butylphenyl)trimethoxysilane.

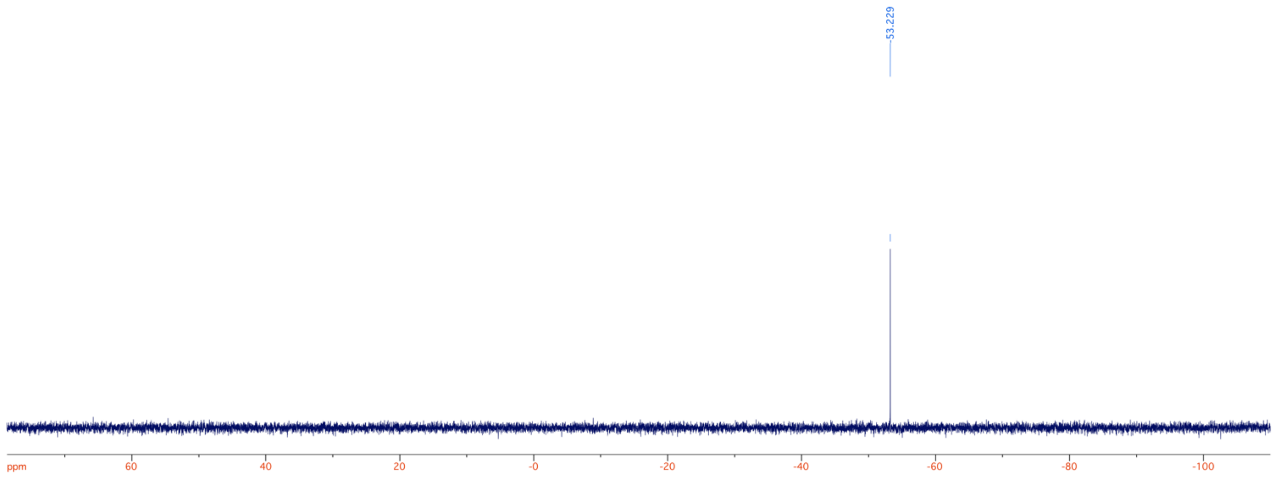

Figure S13. ^{29}SiNMR spectrum of (4-*tert*-butylphenyl)trimethoxysilane.

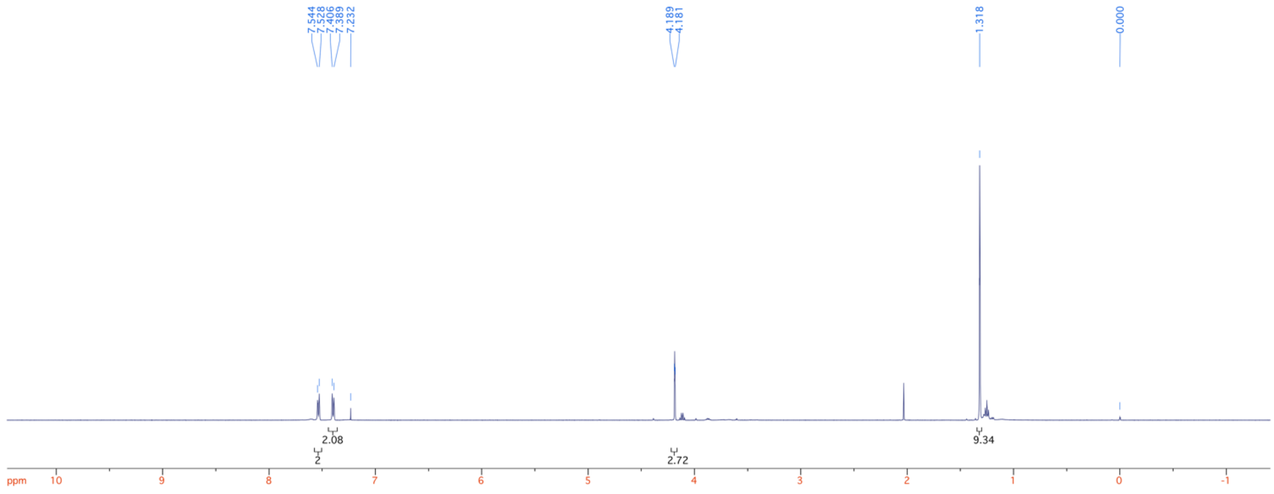

Figure S14. ^{1}H NMR spectrum of (4-*tert*-butylphenyl)silane.

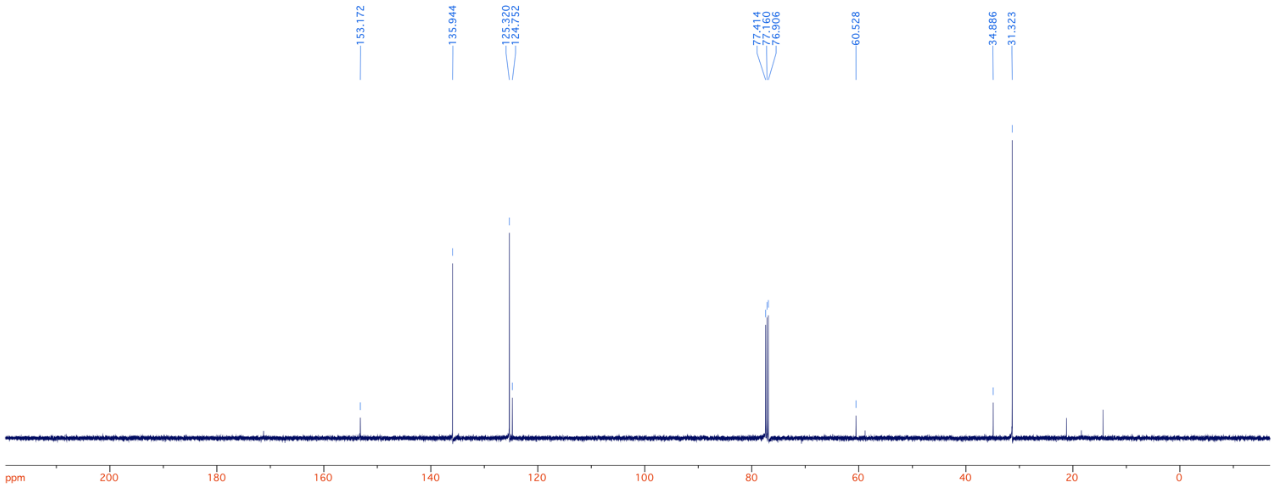

Figure S15. ^{13}C NMR spectrum of (4-*tert*-butylphenyl)silane.

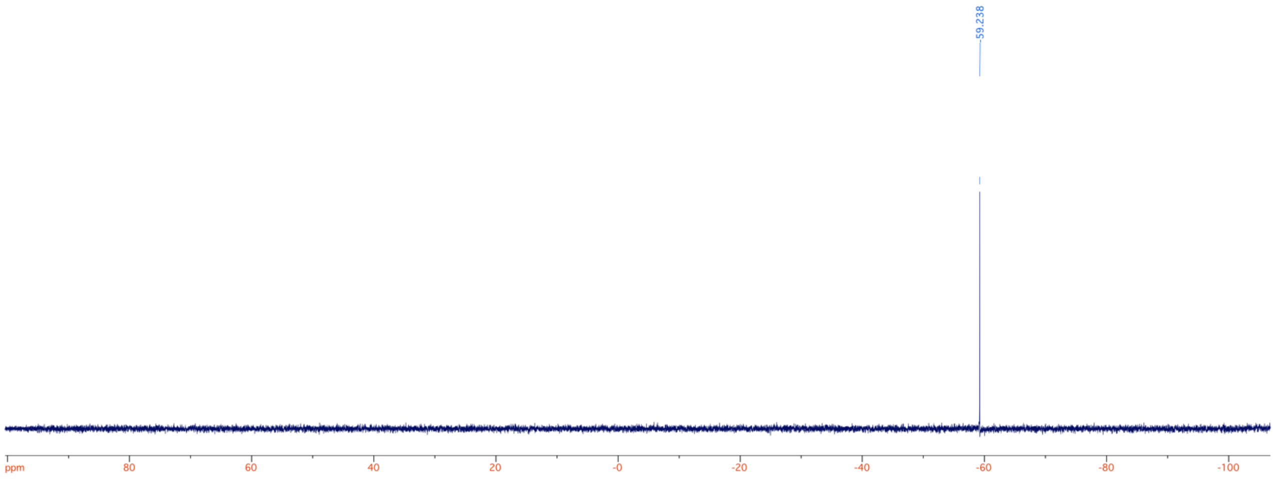

Figure S16. ^{29}Si NMR spectrum of (4-*tert*-butylphenyl)silane.

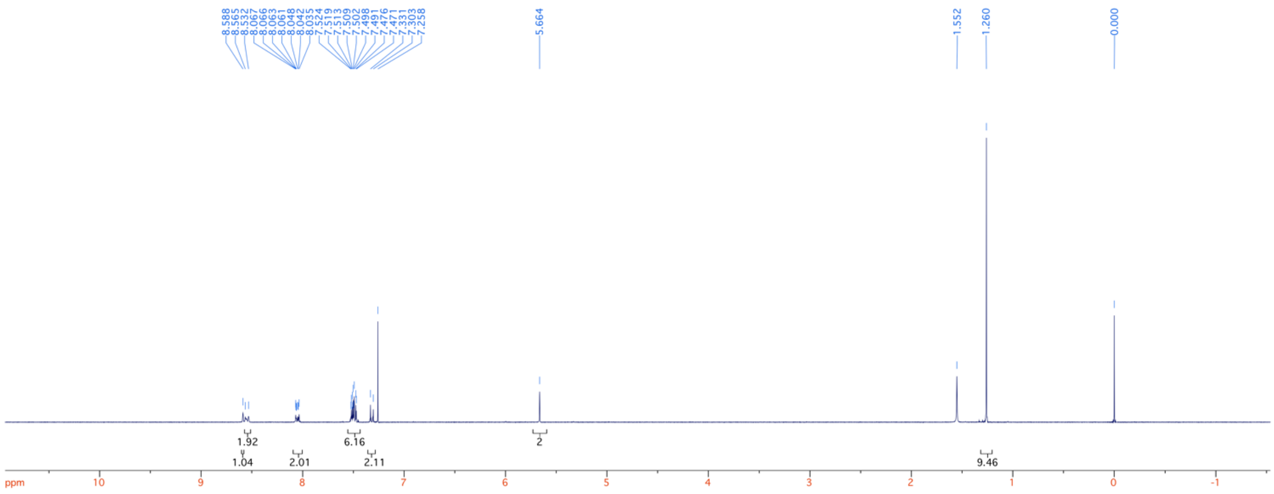

Figure S17. ^{1}H NMR spectrum of **1b**.

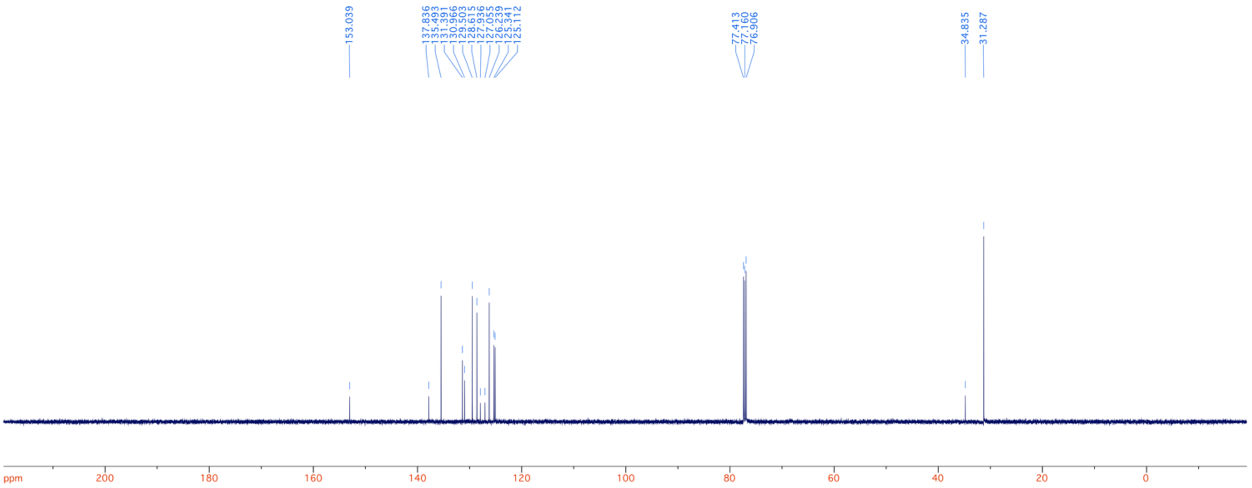

Figure S18. ^{13}C NMR spectrum of **1b**.

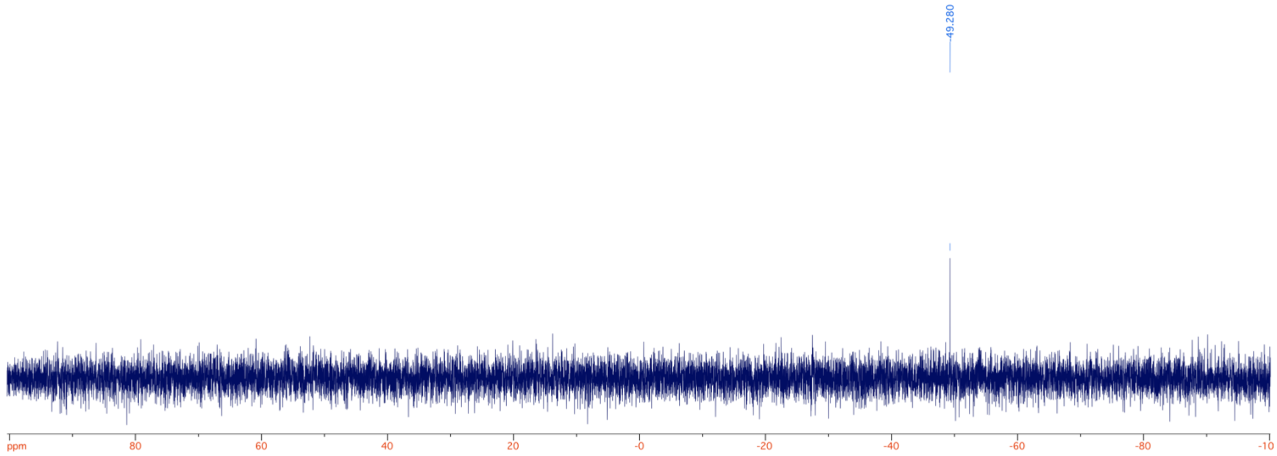

Figure S19. ^{29}Si NMR spectrum of **1b**.

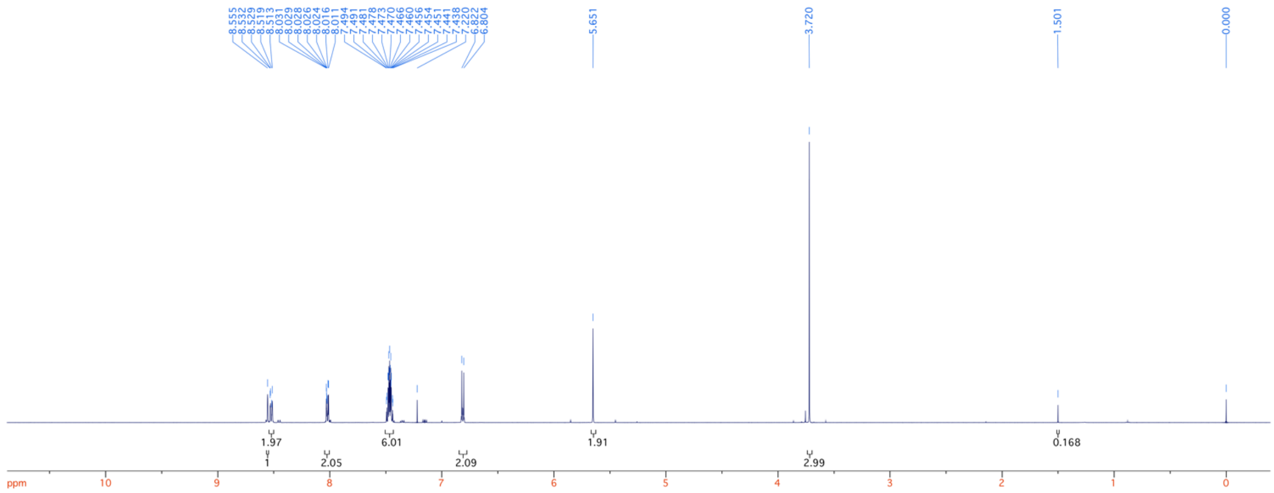

Figure S20. ^{1}H NMR spectrum of **1c**.

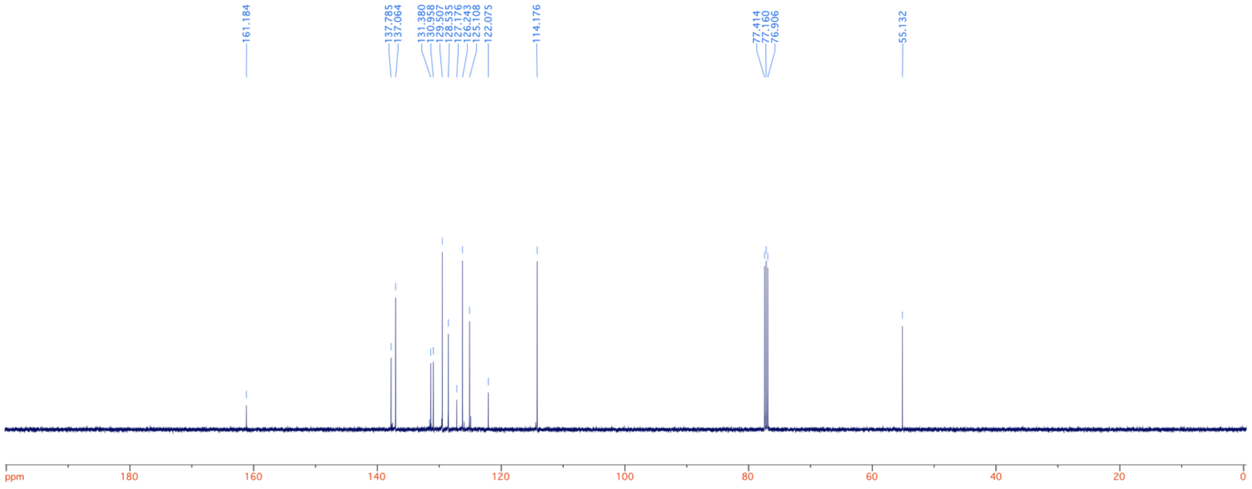

Figure S21. ^{13}C NMR spectrum of **1c**.

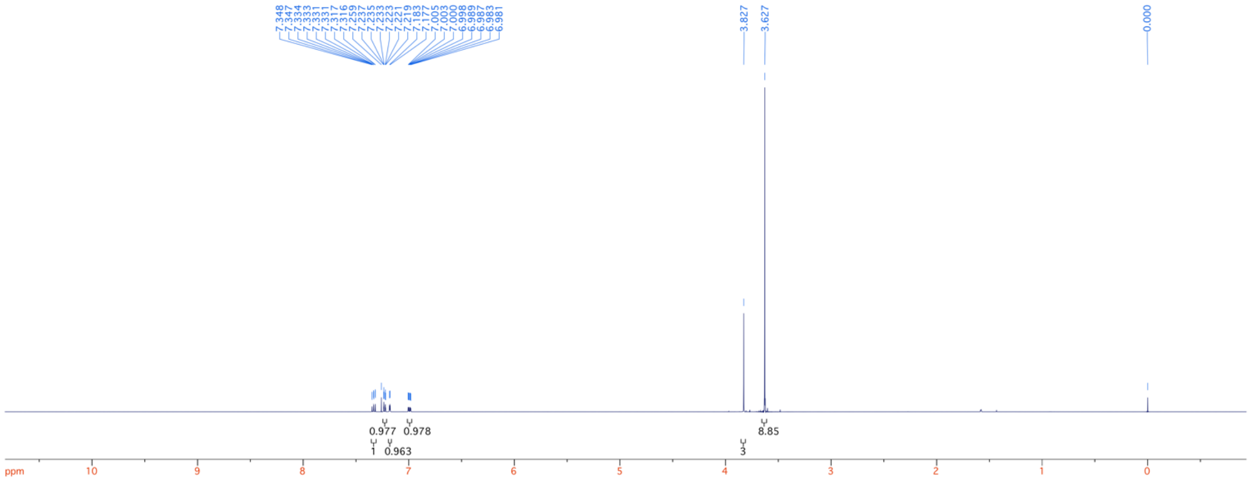

Figure S22. ^{1}H NMR spectrum of (3-methoxyphenyl)trimethoxysilane.

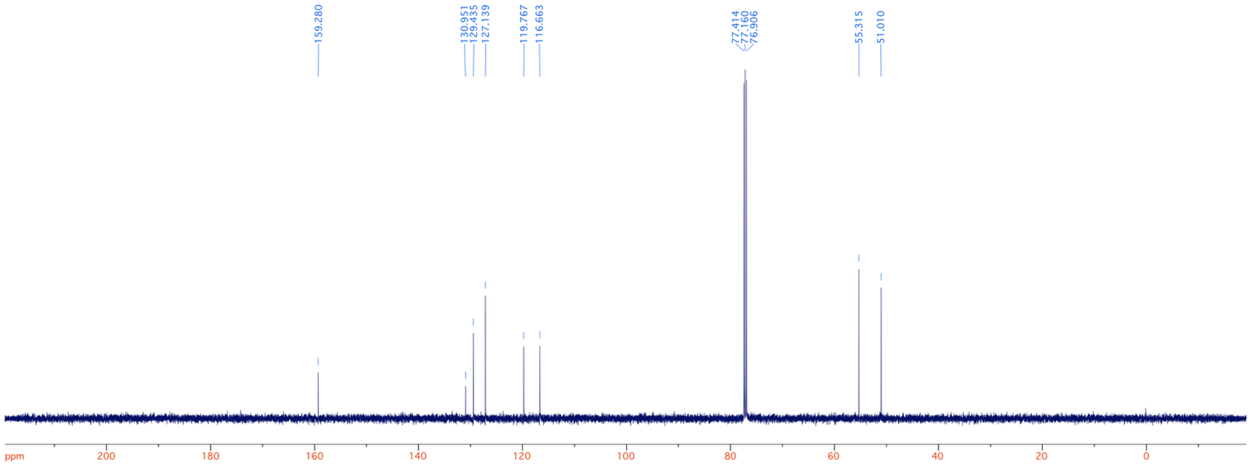

Figure S23. ^{13}C NMR spectrum of (3-methoxyphenyl)trimethoxysilane.

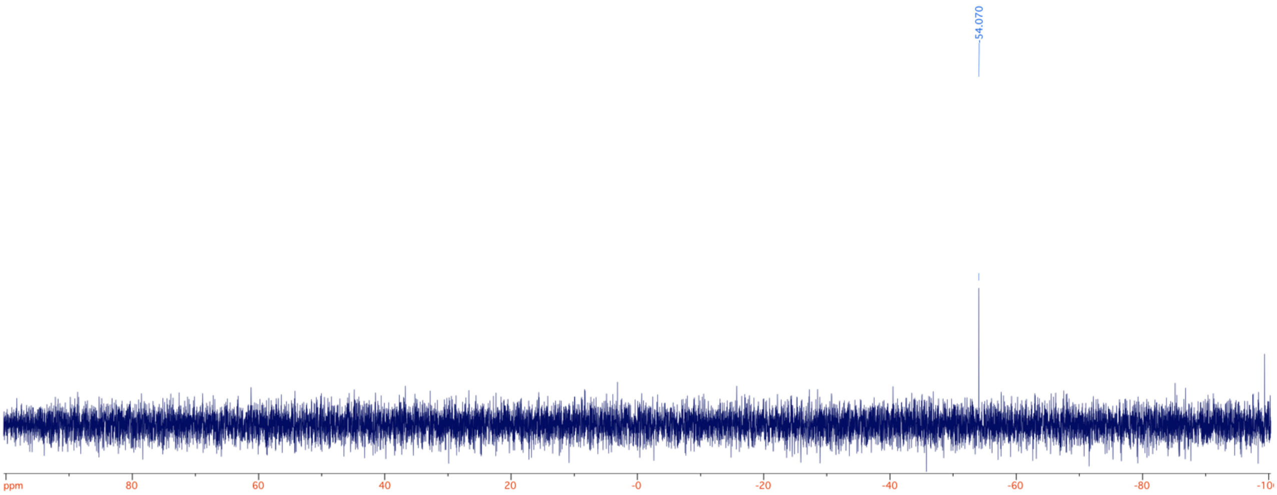

Figure S24. ^{29}SI NMR spectrum of (3-methoxyphenyl)trimethoxysilane.

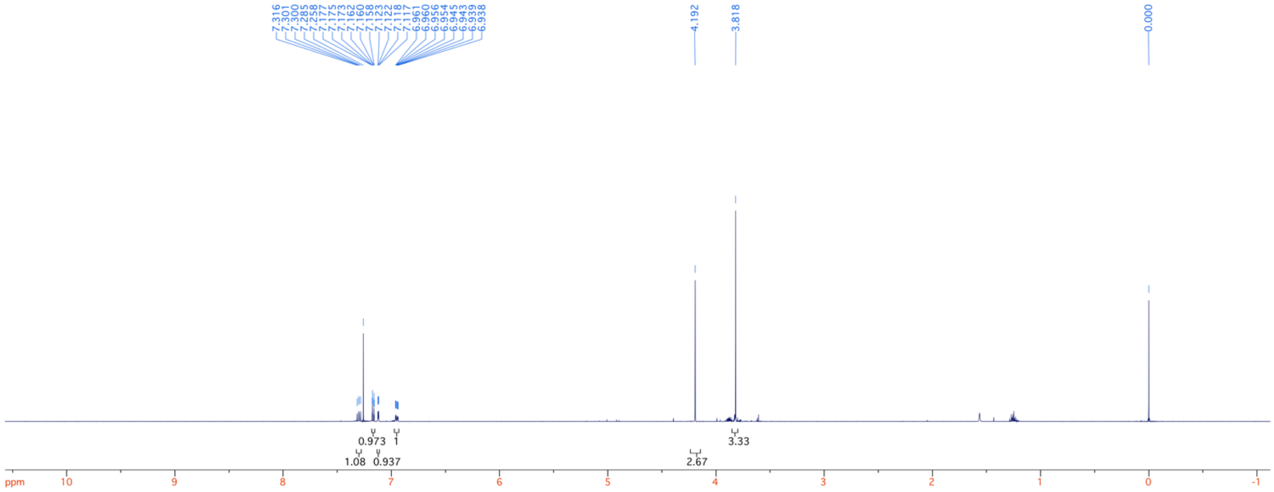

Figure S25. ^{1}H NMR spectrum of (3-methoxyphenyl)silane.

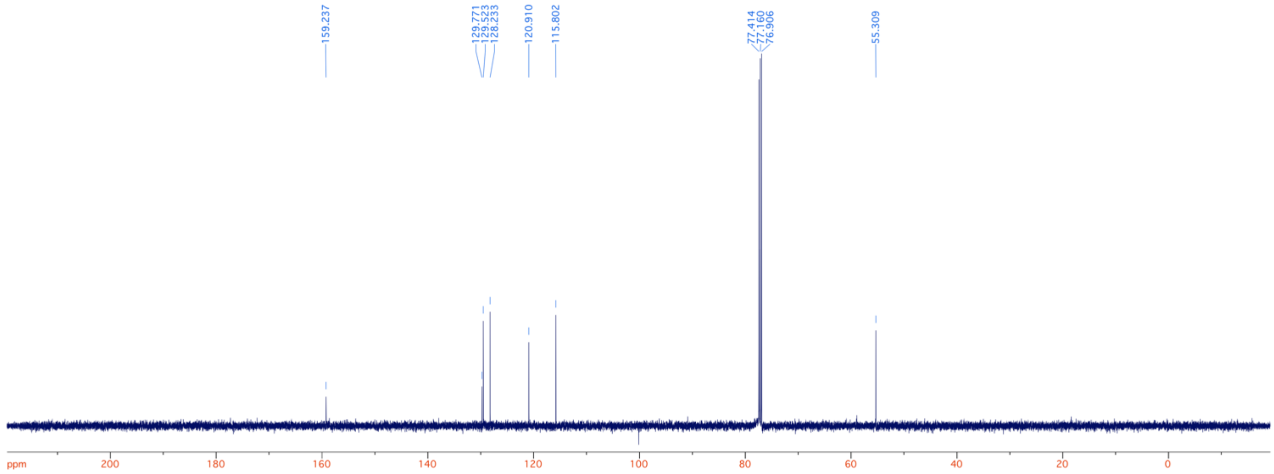

Figure S26. ^{13}C NMR spectrum of (3-methoxyphenyl)silane.

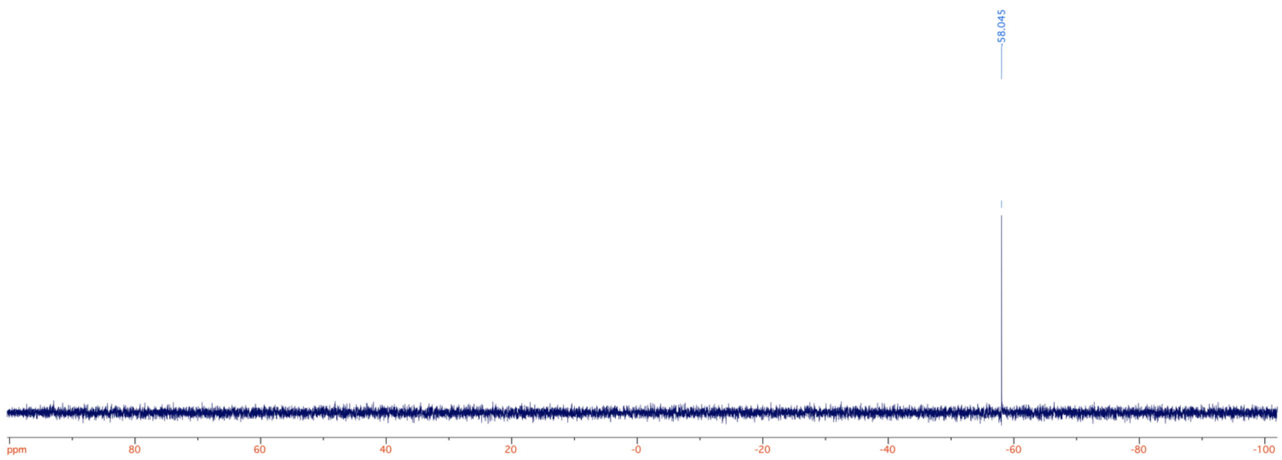

Figure S27. ^{29}Si NMR spectrum of (3-methoxyphenyl)silane.

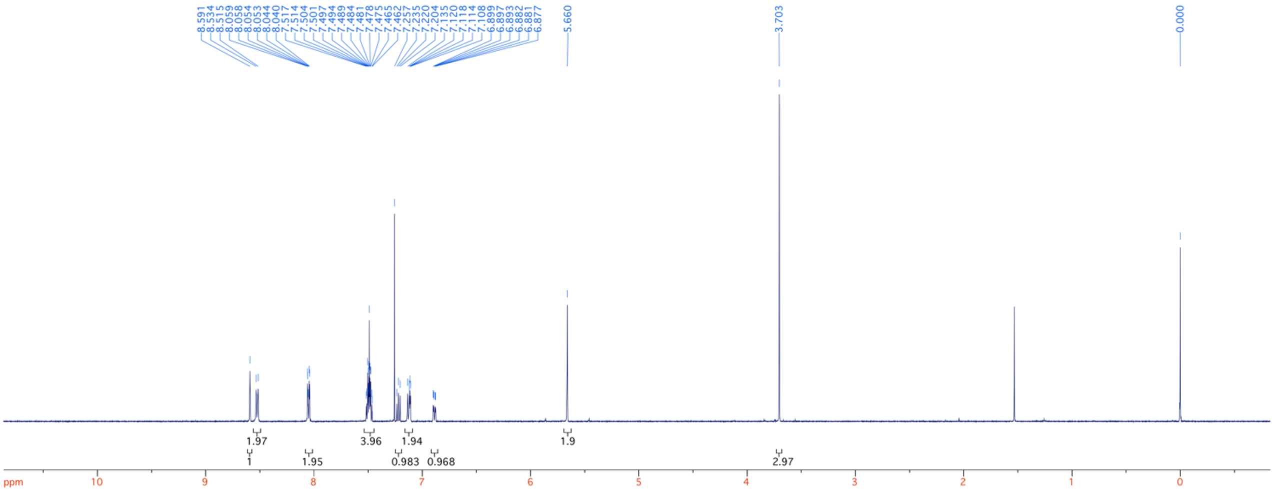

Figure S28. ^{1}H NMR spectrum of **1d**.

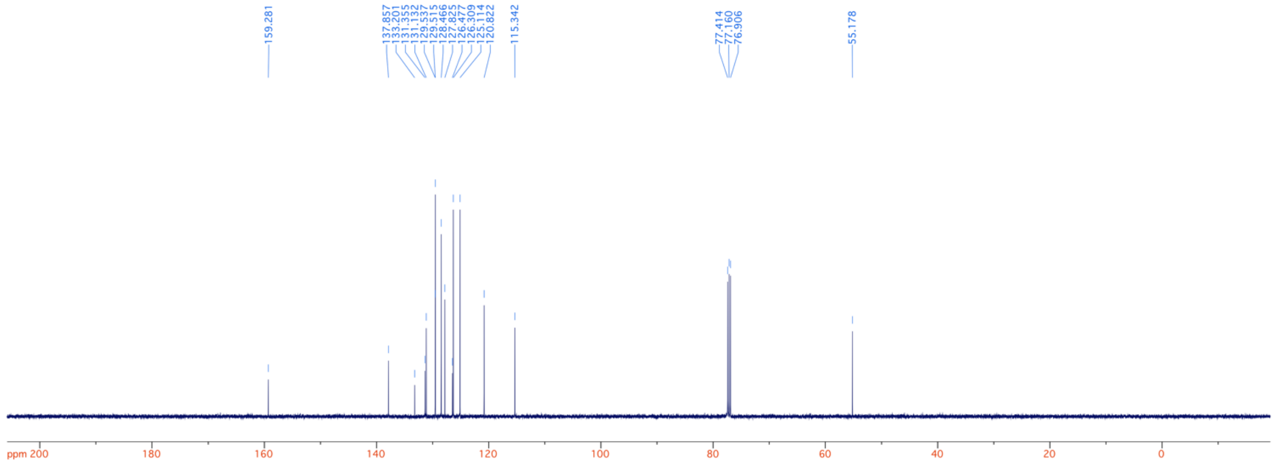

Figure S29. ^{13}C NMR spectrum of **1d**.

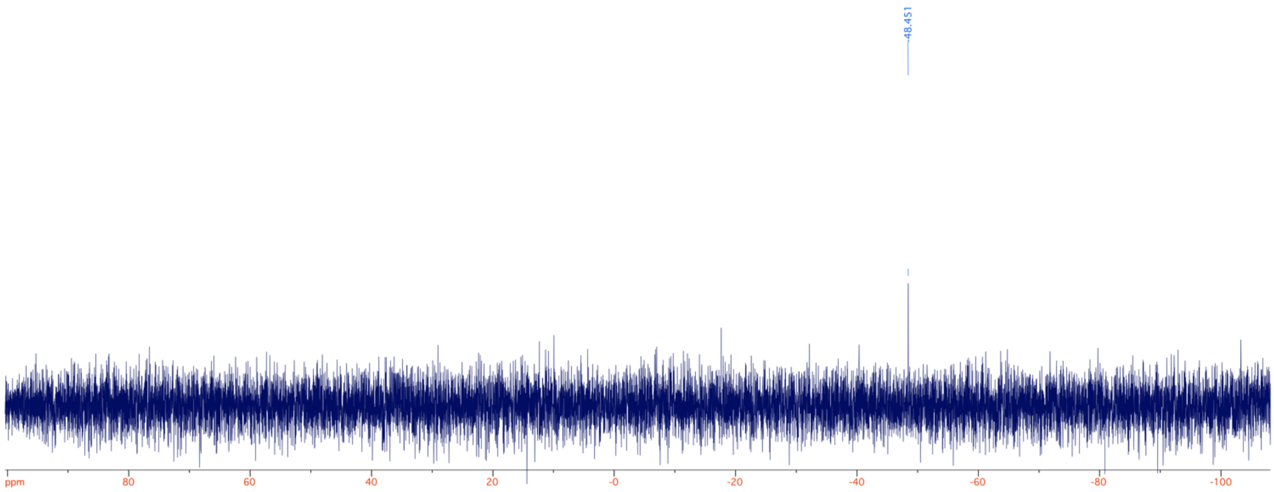

Figure S30. ^{29}Si NMR spectrum of **1d**.

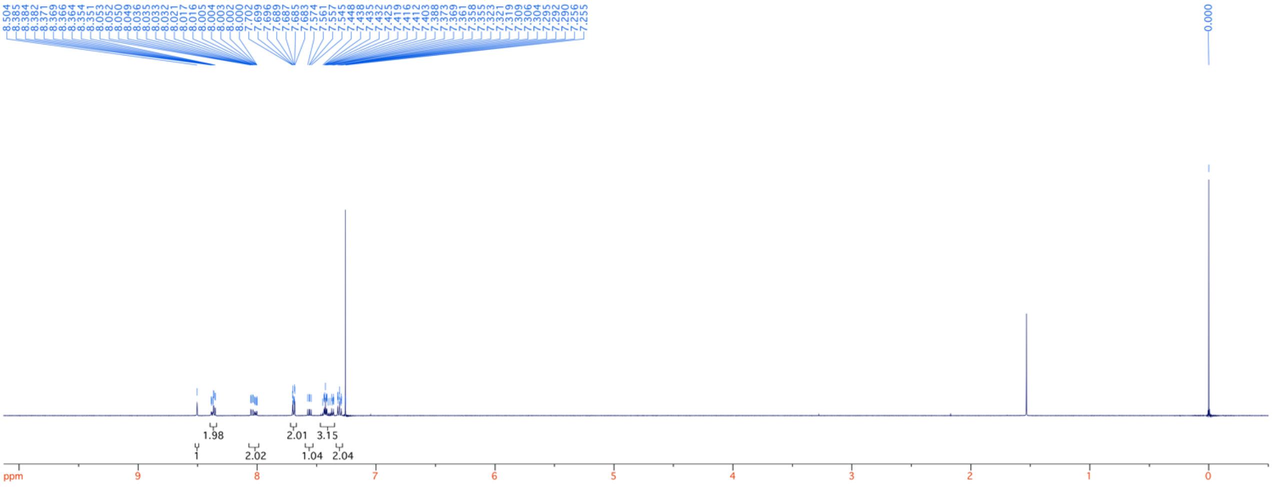

Figure S31. ^{1}H NMR spectrum of **2a**.

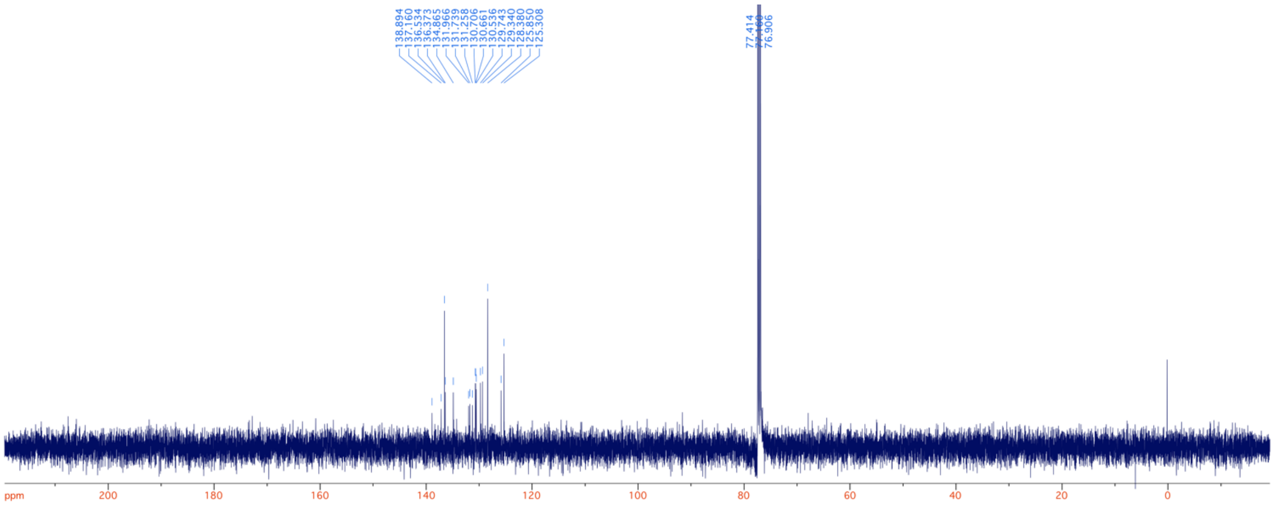

Figure S32. ^{13}C NMR spectrum of **2a**.

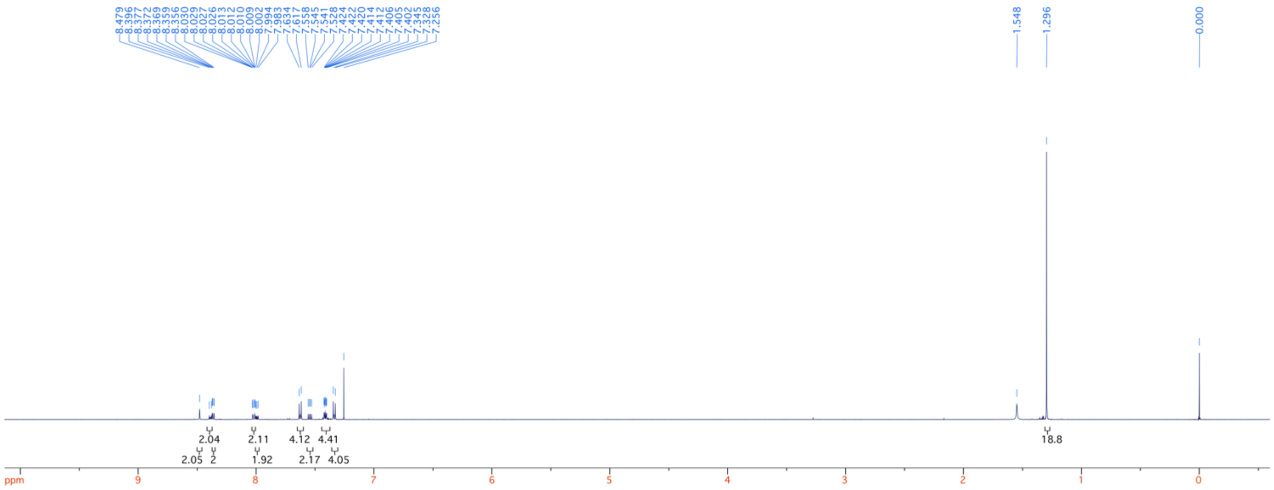

Figure S33. ^{1}H NMR spectrum of **2b**.

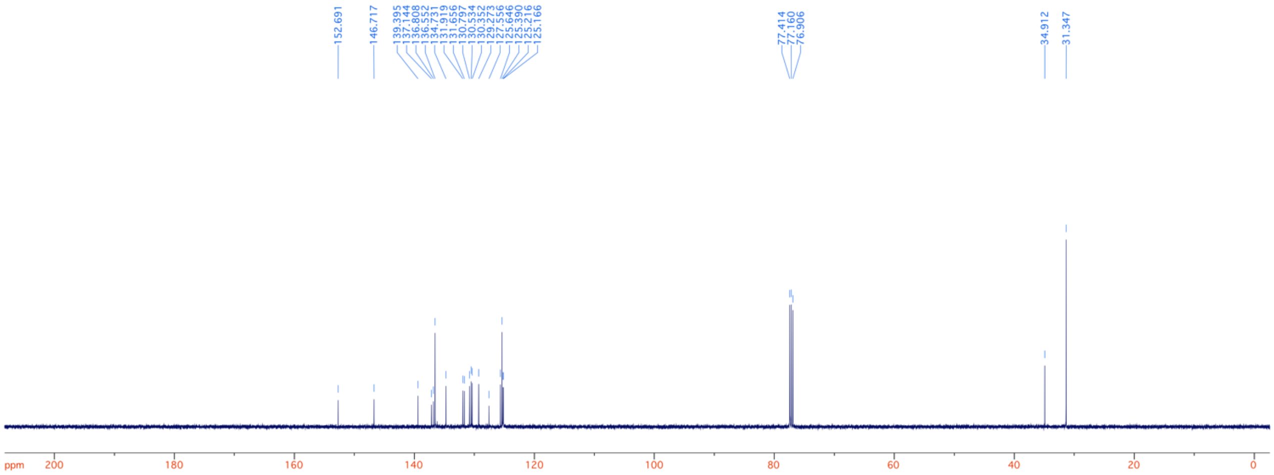

Figure S34. ^{13}C NMR spectrum of **2b**.

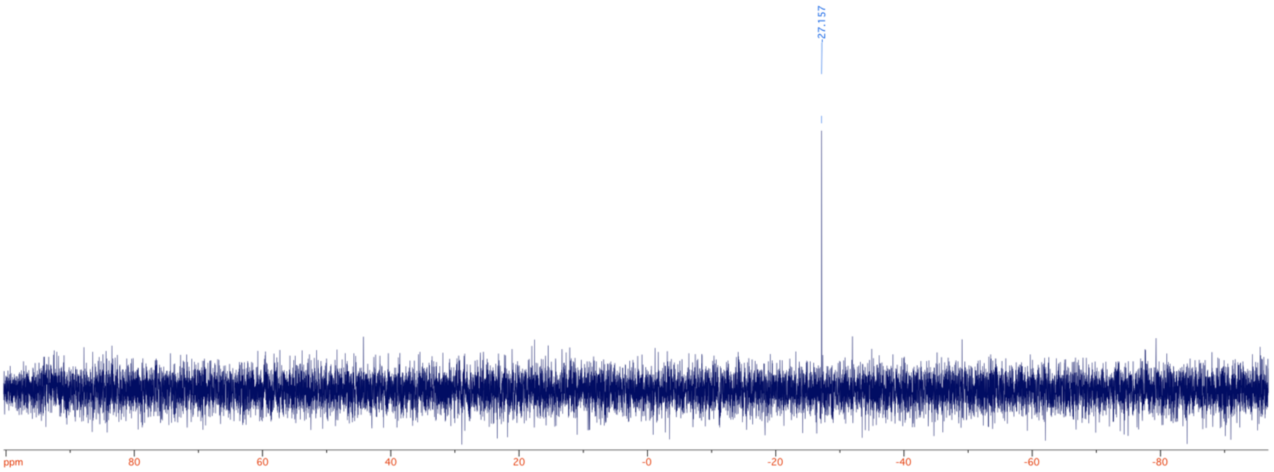

Figure S35. ^{29}Si NMR spectrum of **2b**.

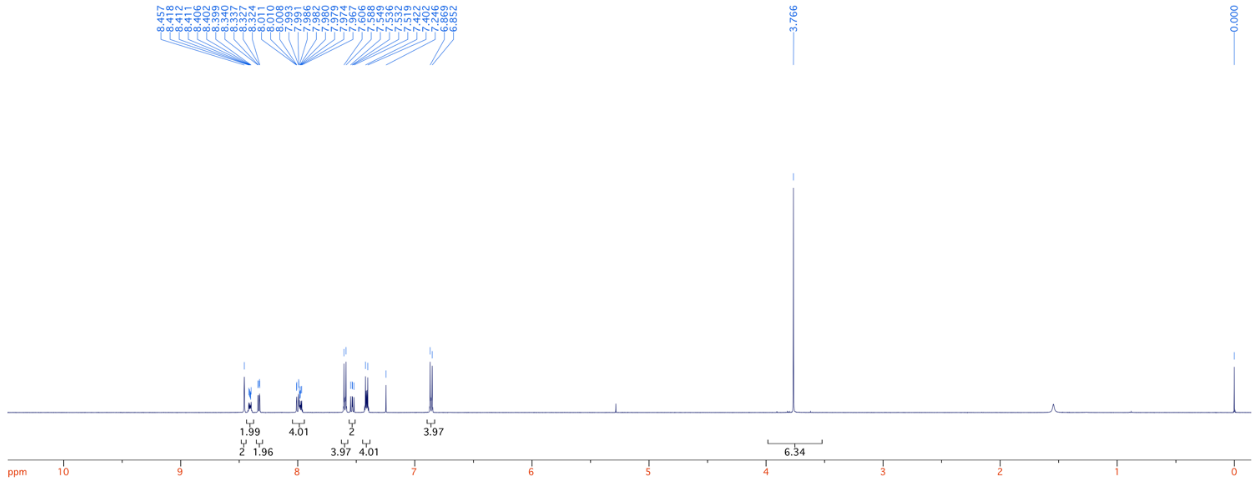

Figure S36. ^{1}H NMR spectrum of **2c**.

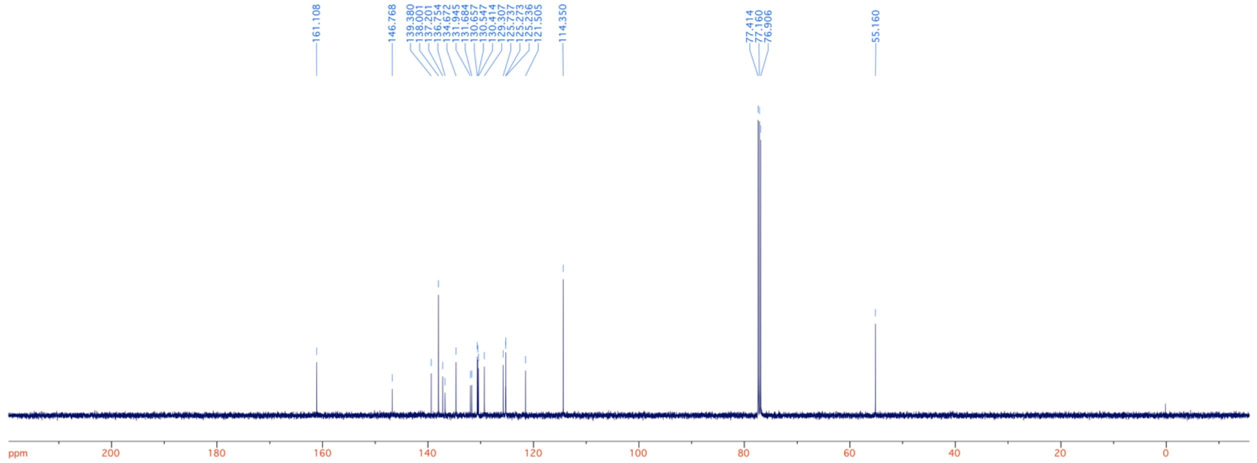

Figure S37. ^{13}C NMR spectrum of **2c**.

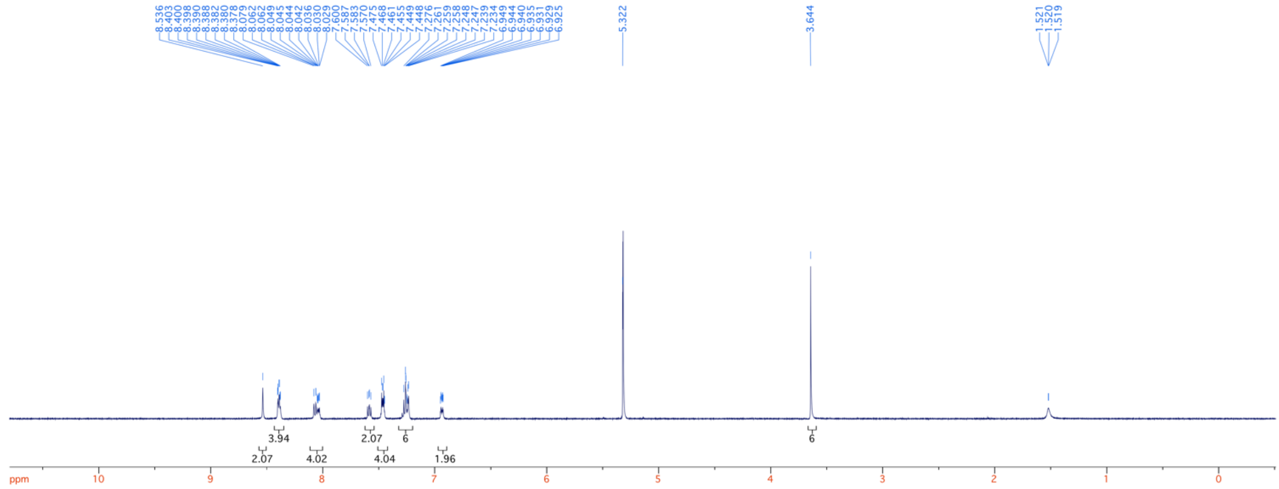

Figure S38. ^{1}H NMR spectrum of **2d**.

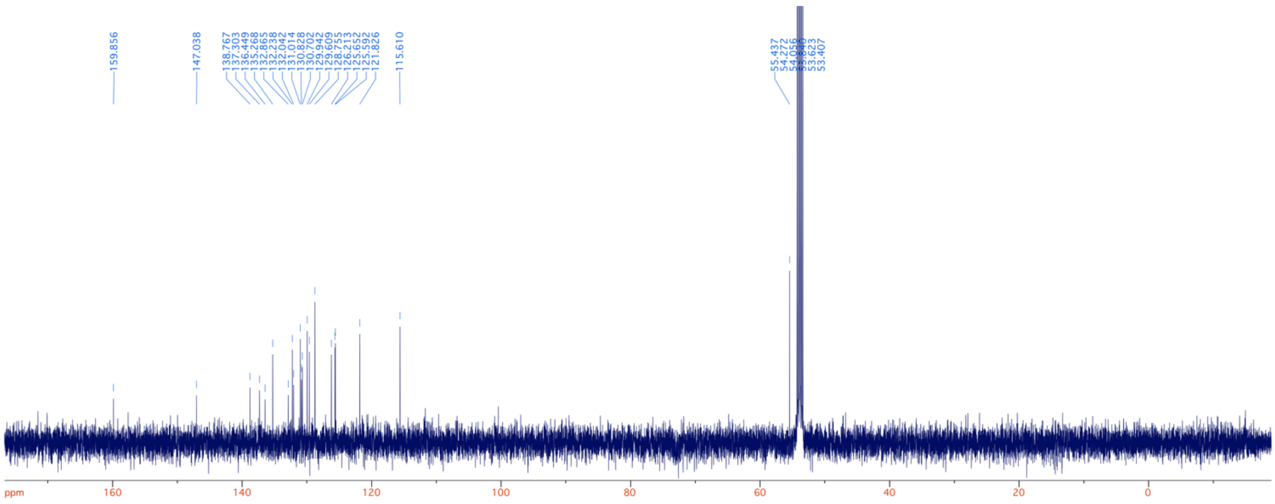

Figure S39. ^{13}C NMR spectrum of **2d**.

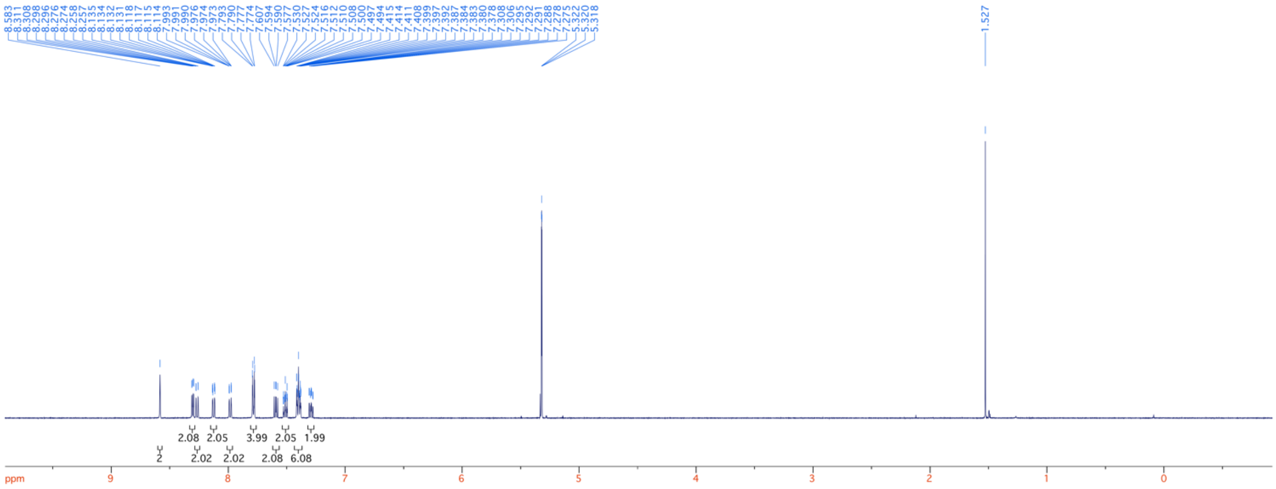

Figure S40. ^{1}H NMR spectrum of **3a**.

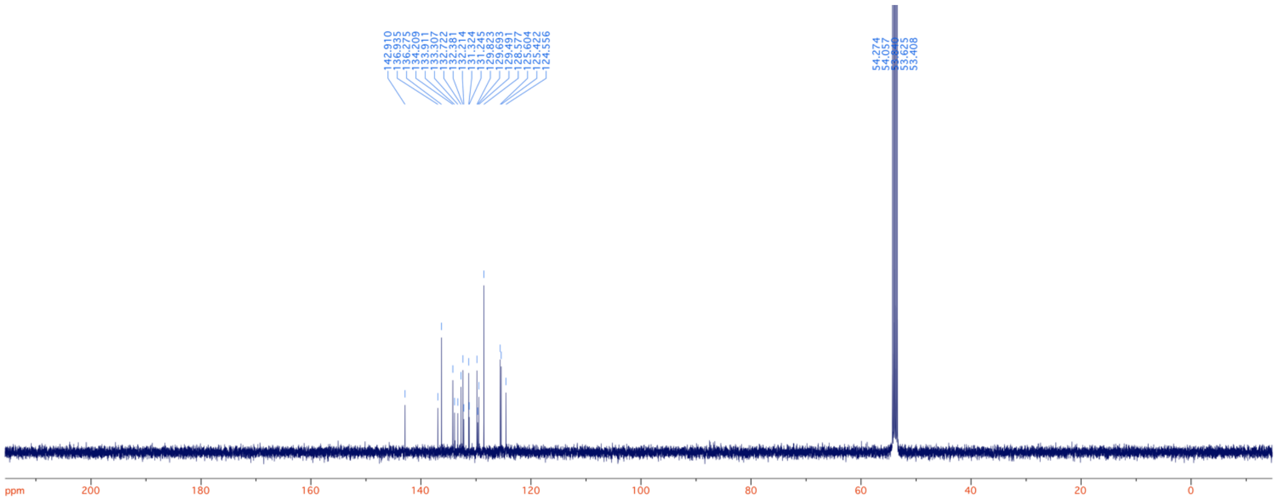

Figure S41. ^{13}C NMR spectrum of **3a**.
